# Native mass spectrometry reveals the conformational diversity of the UVR8 photoreceptor

**DOI:** 10.1073/pnas.1813254116

**Published:** 2019-01-04

**Authors:** Inês S. Camacho, Alina Theisen, Linus O. Johannissen, L. Aranzazú Díaz-Ramos, John M. Christie, Gareth I. Jenkins, Bruno Bellina, Perdita Barran, Alex R. Jones

**Affiliations:** ^a^School of Chemistry, The University of Manchester, Manchester M13 9PL, United Kingdom;; ^b^Photon Science Institute, The University of Manchester, Manchester M13 9PL, United Kingdom;; ^c^Manchester Institute of Biotechnology, The University of Manchester, Manchester M1 7DN, United Kingdom;; ^d^Biometrology, Division of Chemical Medical and Environmental Sciences, National Physical Laboratory, Teddington TW11 0LW, United Kingdom;; ^e^Institute of Molecular, Cell and Systems Biology, College of Medical, Veterinary and Life Sciences, University of Glasgow, Glasgow G12 8QQ, United Kingdom

**Keywords:** UVR8, native mass spectrometry, ion mobility, plant photoreception, intrinsically disordered proteins

## Abstract

UVR8 is a plant photoreceptor protein that regulates photomorphogenic and protective responses to UV light. The inactive, homodimeric state absorbs UV-B light, resulting in dissociation into monomers, which are considered to be the active state and comprise a β-propeller core domain and intrinsically disordered N- and C-terminal tails. The C terminus is required for functional binding to signaling partner COP1. To date, however, structural studies have only been conducted with the core domain where the terminal tails have been truncated. Here, we report structural investigations of full-length UVR8 using native ion mobility mass spectrometry adapted for photoactivation. We show that, while truncated UVR8 photoconverts from a single conformation of dimers to a single monomer conformation, the full-length protein exists in numerous conformational families. The full-length dimer adopts both a compact state and an extended state where the C terminus is primed for activation. In the monomer the extended C terminus destabilizes the core domain to produce highly extended yet stable conformations, which we propose are the fully active states that bind COP1. Our results reveal the conformational diversity of full-length UVR8. We also demonstrate the potential power of native mass spectrometry to probe functionally important structural dynamics of photoreceptor proteins throughout nature.

## Significance

The plant photoreceptor UVR8 absorbs UV-B light to regulate UV protection and photomorphogenic responses in plants. Here we show that UVR8 adopts multiple conformations to generate the signaling active state. The conformational diversity of UVR8 was revealed using a native mass spectrometry approach, where the photoreceptor was photoactivated in the ion source. Our analyses show that not only disordered but also ostensibly well-folded regions of UVR8 can adopt highly extended conformations that are likely to enhance interactions with partner proteins to facilitate signal propagation. The methodology employed could be used to investigate the structural dynamics of other proteins that are activated by light.

**Fig. 1.**
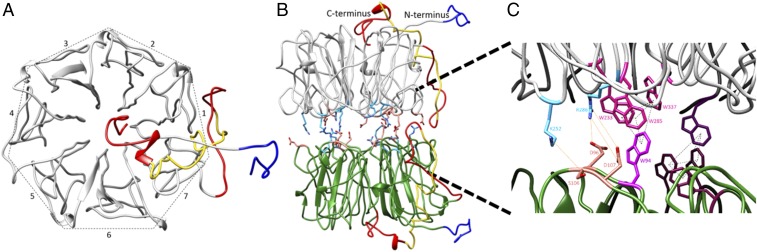
Structural features of the UVR8 photoreceptor. We have modeled in the conformationally disordered C- (red) and N- (dark blue) terminal tails onto the published (10) structure of the UVR8 core domain (see *SI Appendix* and *Methods* for details). The C27 region of the C terminus required for binding to COP1 (6) is highlighted in yellow. (*A*) Top view of a UVR8 monomer, showing the WD40, β-propeller structure of the core domain, and the relative positions of the C- and N-terminal tails. The seven blades of the propeller (numbered 1–7) have a nearly identical main-chain conformation, each blade is contiguous in sequence, and the N- and C-terminal tails emanate from the first (blade 1) and last (blade 7), respectively. (*B*) Side view of the UVR8 homodimer, which is stabilized by cross-dimer salt bridges (positive residues, light blue; negative residues, peach). For consistency, the core domain in each monomer is colored gray and green, respectively; this color scheme aids with clarity in subsequent figures. (*C*) The UVR8 dimer contains two chromophores (pink/purple), each of which comprises a pyramid of tryptophan residues: a triad from one monomer and a fourth from the other. Each pyramid neighbors residues involved in key cross-dimer salt bridges (shown for the pink chromophore).

The functional importance of disorder in proteins is transforming the structure–function paradigm. Intrinsically disordered regions (IDRs) of proteins (1) have been found to play a particularly important role in cell-signaling pathways, where the lack of structural order provides accessible and often versatile sites for binding to signaling partners and for posttranslational modification (2). Consistent with this, it is becoming increasingly apparent that IDRs facilitate signal propagation following activation of photoreceptor proteins by environmental light cues (e.g., refs. 3–7). The plant photoreceptor UV RESISTANCE LOCUS8 (UVR8) (8, 9) serves as an illustrative example. It comprises a structurally well-defined seven-blade β-propeller core domain (10, 11) alongside what are predicted to be conformationally flexible C- and N-terminal tails (Fig. 1 *A* and *B* and *SI Appendix*, Fig. S1) (10). In the dark UVR8 adopts a homodimeric structure, which is stabilized by cross-dimer salt bridges (Fig. 1*B*). Absorption of UV-B light (280–315 nm) by chromophores made up of clusters of tryptophan residues at the dimer interface (Fig. 1*C*) results in disruption of these salt bridges, which in turn leads to dissociation into monomers (10–12) and accumulation of UVR8 in the nucleus (13). Although it is widely accepted that monomerization is necessary for UVR8 activation, it is now clear that the C-terminal tail is vital for subsequent signal transduction and for regulation of UVR8 function (6, 12, 14).

In the nucleus of the cell, photoactivated UVR8 monomer binds to CONSTITUTIVELY PHOTOMORPHOGENIC1 (COP1), the central regulator of light signaling in plants (12, 15). Together they regulate over 100 genes involved in UV-B acclimation (e.g., biosynthesis of UV-absorbing flavonoids) and photomorphogenic responses such as suppression of hypocotyl growth (15). The C terminus contains a highly conserved sequence of 27 amino acids (aa) (residues 397–423 of 440) that is known to be required not only for functional binding to COP1 but is also necessary and sufficient for binding to the REPRESSOR OF UV-B PHOTOMORPHOGENESIS proteins, RUP1 and RUP2, which are negative regulators of UVR8 (6, 16). Indeed, expression of just the final 44 aa of the C terminus (residues 397–440) in plants is sufficient to trigger expression of the transcription factor ELONGATED HYPOCOTYL5 (HY5) (16), which mediates many of the responses triggered by UVR8 (14). Despite these data supporting a functional role for the C-terminal IDR, little is known about the extent of disorder of the C terminus or about its structural dynamics and how they relate to the active state of UVR8. Crystal structures have been solved for the UVR8 core domain (residues 12–381, UVR8^12–381^) (10, 11, 17) and have yielded significant mechanistic information regarding light-induced monomerization. In order for the protein to crystallize, however, it was necessary to truncate the N- and C-terminal tails, both implying structural disorder in these regions of UVR8 and precluding their detailed study. Here, we have acquired data of both UVR8^12–381^ and full-length UVR8 using native ion mobility mass spectrometry instrumentation that incorporates illumination of the sample solution at the ion source. These data not only enable monitoring of the UV-B–induced dissociation of the UVR8 dimer but also reveal the conformational diversity of the full-length protein. We believe this method will prove to be a widely applicable and powerful technique with which to investigate both photoreceptor activation mechanisms and their signaling conformations.

## Results

### A Simple Optical Assembly Enables the Investigation of Photoreceptor Structural Dynamics by Native Ion Mobility Mass Spectrometry.

**Fig. 2.**
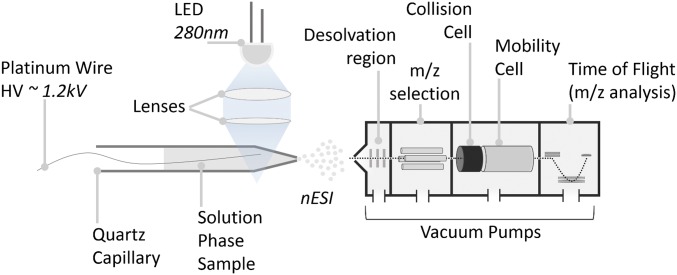
Photo-ion mobility mass spectrometry. Schematic of the experimental setup. The liquid phase sample is loaded in a quartz capillary (10 µL). Using a lens assembly the light emitted from a LED is focused at the end of the capillary. High voltage is applied to the solution, resulting in the nebulization of the sample and its transfer to the gas phase by nESI. The generated ions are then transferred into an ion mobility mass spectrometer where CCS and molecular weight (*m/z*) data are obtained.

Native ion mobility mass spectrometry was used to investigate the photoactivation of UVR8 using a simple, versatile, and modular illumination assembly (Fig. 2). A high-power LED (25 mW, 350 mA; Thorlabs) emitting at a peak wavelength of 280 nm was mounted above the ion source. The output was focused onto the nanoelectrospray ionization (nESI) tip of the ion source using convex lenses, allowing irradiation of the sample solution before desolvation and before it enters the mass spectrometer. This illumination assembly is compatible with a range of commercial spectrometers and has been used with spectrometers from three different manufacturers in this study (*Methods*). All known photoreceptor proteins are activated with wavelengths corresponding to the solar emission spectrum that reaches the Earth’s surface. This range (near UV–visible–near IR) is covered by inexpensive and readily available LEDs similar to the one used here. In principle, therefore, the illumination assembly can be straightforwardly adapted for investigation of the structural changes that follow photoactivation of all light-responsive proteins—and indeed many other photochemical reactions.

Ion mobility spectrometry measures the movement of ions in a drift cell through an inert buffer gas in the presence of a weak electric field. The electric field axially drives ions through the drift cell while the buffer gas retards their motion via numerous collisions. The result is a temporal separation of ions based upon the interaction of that ion with the gas, which in turn is dependent on its shape and charge (z), which is recorded as an arrival time distribution. The arrival time distribution of any given ion can be converted to a collision cross-section (CCS) distribution (18). The shape of this distribution for a species with a given nominal *m/z* is made up of the expected diffusion of the initial ion pulse as the ions traverse the cell as well as contributions from conformers or *m/z* coincident species, which may be higher-order oligomers or isomers. In general, a narrow drift time peak often relates to a compact and well-defined structure where broader distributions reveal the presence of more conformers and a more dynamic structure in solution.

### The UVR8 Core Domain Exists in a Single Conformational Family.

**Fig. 3.**
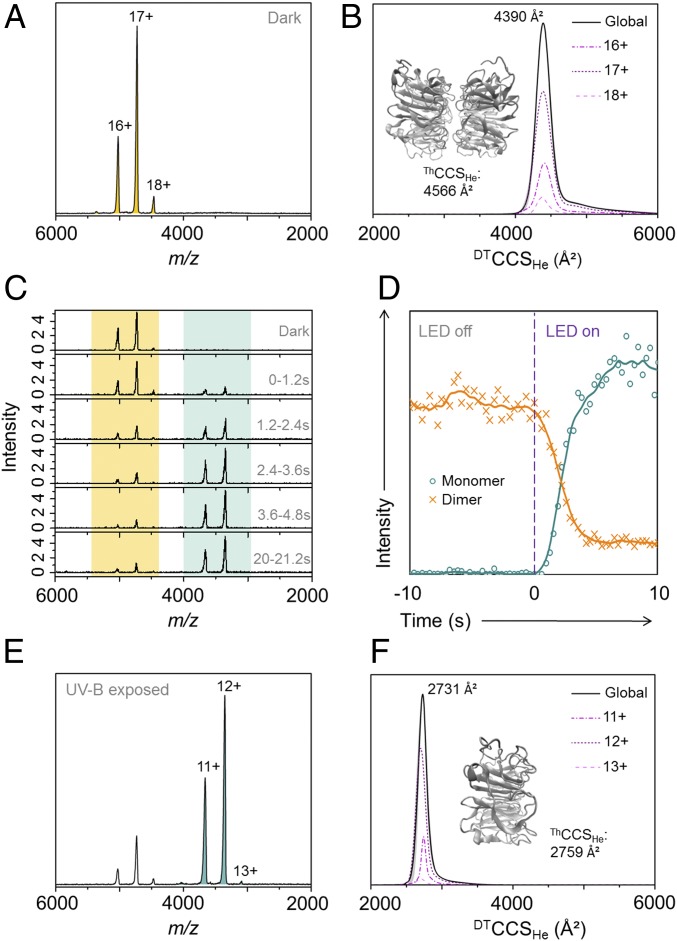
Native mass spectral and ion mobility data of the UVR8 core domain (UVR8^12–381^). The mass spectra (*A*, *C*, and *E*) and ion chromatogram (*D*) in this figure were acquired using an Ultima Global mass spectrometer, and the ^DT^CCS_He_ (*B* and *F*) were acquired using a Synapt G2 modified with a linear drift tube. (*A*) Native mass spectrum of the UVR8^12–381^ dimer (pale-orange peaks) in the absence of UV-B light with charge states labeled. (*B*) The monodisperse ^DT^CCS_He_ of the UVR8^12–381^ dimer measured in helium. (*Inset*) Energy-minimized structure of the UVR8^12–381^ dimer and associated ^Th^CCS_He_. (*C*) Mass spectra of UVR8^12–381^ as a function of illumination time using a high-power LED (280 nm, 25 mW, 350 mA). Mass spectra were combined over a period of 1.2 s after the LED was switched on at *t*_0_. Dimer signal, pale orange; monomer signals, pale green. (*D*) Ion chromatogram extracted for the UVR8^12–381^ dimer (orange) and monomer (green) as a function of data acquisition time. Data to the left of the purple dashed line are from when the ion source tip is in the dark and the data to the right are from when the tip is illuminated. (*E*) Native mass spectrum of UVR8^12–381^ following illumination in the source tip with the 280-nm LED for 10 s (to ensure maximum conversion). Residual dimer peaks are still evident, but the spectrum is now dominated by the UVR8^12–381^ monomer (pale-green peaks). (*F*) The monodisperse ^DT^CCS_He_ of the UVR8^12–381^ monomer measured in helium. (*Inset*) Energy-minimized structure of the UVR8^12–381^ monomer and associated ^Th^CCS_He_.

Crystal structures have been solved for UVR8^12–381^ where the N- and C-terminal tails were truncated from the full-length protein (10, 11, 17). To investigate the conformational flexibility of UVR8 in the absence of its IDRs we first acquired the native mass spectrum of the UVR8^12–381^ dimer in the absence of UV-B light (Fig. 3*A*). These data reveal a narrow charge-state distribution where z = 15+ to 18+, consistent with the UVR8^12–381^ dimer existing in a single conformational family. This conclusion is supported by ion mobility drift time data acquired in both helium (Fig. 3*B*) and nitrogen (*SI Appendix*, Fig. S2*A*). A global CCS, ^DT^CCS_He_, of 4,390 ± 3 Å^2^ was obtained from these data and is in good agreement with the theoretical value, ^Th^CCS_He_, of 4,566 ± 43 Å^2^ from molecular dynamics (MD) simulations (*SI Appendix*, Fig. S3*A*) of the energy-minimized, published (10) structure (Fig. 3*B*, *Inset*). From these data we conclude that the UVR8^12–381^ dimer is structurally ordered.

When UV-B light from the 280-nm LED illuminates the UVR8^12–381^ dimer in solution throughout data acquisition, the intensity of the UVR8^12–381^ dimer signal decreases with a concomitant appearance and increase in intensity of signal from the UVR8^12–381^ monomer (Fig. 3*C*). The dimer signal decreases in intensity up to 3.6–4.8 s of data acquisition, after which it remains constant. The monomer can be seen after up to 1.2 s and increases in intensity up to 3.6–4.8 s. The fact that depletion of the UVR8^12–381^ dimer signal is correlated with the appearance of monomer signal is further illustrated by the ion chromatogram for each in Fig. 3*D*. Although data are acquired as a function of time, once the LED is turned on the sample is continuously illuminated during the subsequent acquisition. At this stage of instrument development, therefore, the kinetics of dimer dissociation cannot be straightforwardly extracted from these data. The native mass spectrum of the UVR8^12–381^ monomer is in Fig. 3*E*, which was acquired after the sample was exposed to UV-B in the ion source for 10 s to ensure maximum conversion. The monomer also presents a narrow charge-state distribution (z = 11+ to 13+), which indicates that a single conformational family of UVR8^12–381^ dimers dissociate into a single conformational family of UVR8^12–381^ monomers. This narrow distribution was again confirmed by the ion mobility data acquired in both helium (Fig. 3*F*) and nitrogen (*SI Appendix*, Fig. S2*B*). A global ^DT^CCS_He_ of 2,731 ± 5 Å^2^ was obtained from these data and is in good agreement with the ^Th^CCS_He_ of 2,759 ± 32 Å^2^ from MD simulations (*SI Appendix*, Fig. S3*B*) of the energy-minimized, published (10) structure (Fig. 3*F*, *Inset*). Much like the UVR8^12–381^ dimer, therefore, the UVR8^12–381^ monomer adopts a well-ordered structure in the absence of its IDRs. The native mass spectra of the UVR8^12-381^ dimer and monomer were measured using three mass analysers (Fig. 3 and *SI Appendix*, Fig. S4) all revealing comparable results. In vitro redimerization of the UVR8^12–381^ monomer in the absence of UV-B was also measured by native mass spectrometry (*SI Appendix*, Fig. S5), where it occurred over several hours in a manner similar to that observed previously by SDS/PAGE (10).

### Full-Length UVR8 Exists in Numerous Conformational Families.

**Fig. 4.**
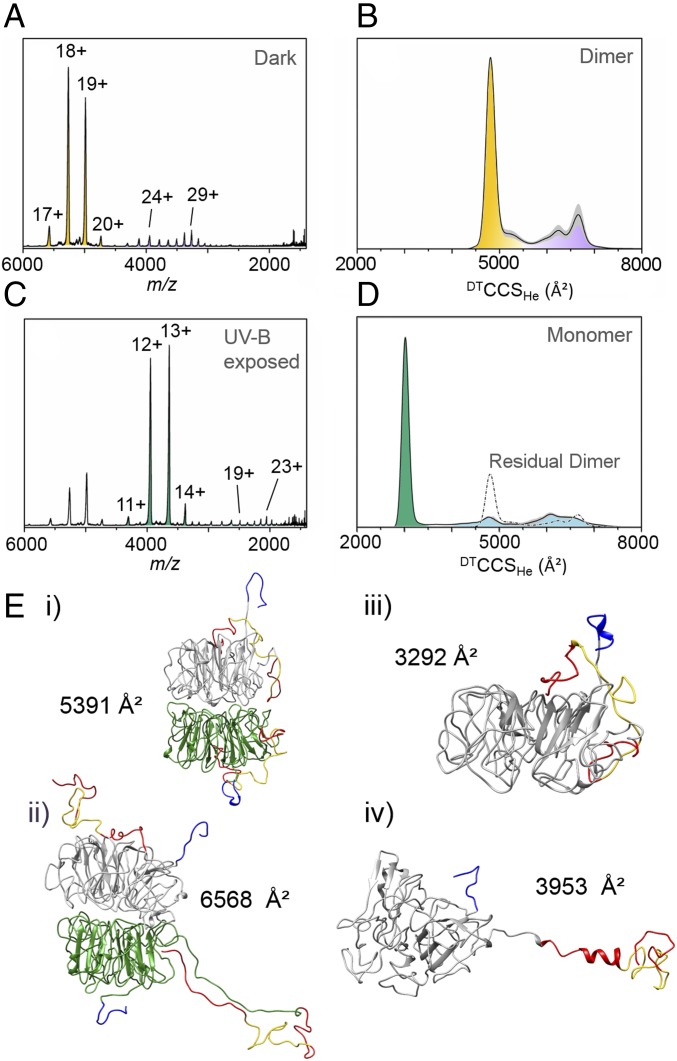
Native mass spectral and ion mobility data of full-length UVR8. All experimental data presented in this figure were acquired using an Agilent 6560 Ion Mobility Q-ToF mass spectrometer. (*A*) Native mass spectrum of the full-length UVR8 dimer in the absence of UV-B light with charge states labeled. The full-length UVR8 dimer presents in at least two different conformational families, compact and extended (orange and purple, respectively). (*B*) ^DT^CCSD_He_ of the full-length UVR8 dimer measured in helium. ^DT^CCSD_He_ of all charge states were weighted according to the intensity of the peak in the mass spectrum and averaged to get the global distribution. The gray shaded areas represent the SD. (*C*) Native mass spectrum of full-length UVR8 following illumination in the source tip with the 280-nm LED for 1 s. Residual dimer peaks are again still evident, but the spectrum is now dominated by the full-length UVR8 monomer, which presents in at least two different conformational families, compact and extended (green and light blue, respectively). (*D*) ^DT^CCS_He_ of the full-length UVR8 monomer measured in helium. The residual dimer signal is represented by a dotted-dashed line. (*E*) Representative structures of full-length UVR8 from gas phase MD simulations: (*i*) compact dimer, (*ii*) extended dimer, (*iii*) compact monomer, and (*iv*) extended monomer. The UVR8 core domains are colored gray and green, the N termini dark blue, the C termini red, and the C27 region yellow.

As we have seen, photoactivation of UVR8^12–381^ results in a straightforward conversion between a single conformational family of structurally ordered dimers to a single conformational family of structurally ordered monomers. In sharp contrast to this, the full-length protein adopts numerous conformational states, both before and after exposure to UV-B. The full-length dimer presents in at least two distributions (Fig. 4*A*), with charge states ranging from z = 17+ to 21+ and from z = 22+ to 31+. Measured ^DT^CCS_He_ values from ion mobility data (Fig. 4*B* and *SI Appendix*, Fig. S6*A*) confirm that these distributions correspond to compact (∼4,500–5,400 Å^2^) and more extended (∼5,600–7,000 Å^2^) forms, respectively. The extended distribution is divided into two subpopulations with the first apex at ^DT^CCS_He_ ∼6,240 Å^2^ and the second at ^DT^CCS_He_ ∼6,660 Å^2^. Because the structure of the core domain appears to be highly stable (i.e., the data of the UVR8^12–381^ dimer in Fig. 3) we hypothesized that these populations correspond to different conformational folds of the C- and N-terminal tails, which are predicted to be disordered (*SI Appendix*, Fig. S1).

**Fig. 5.**
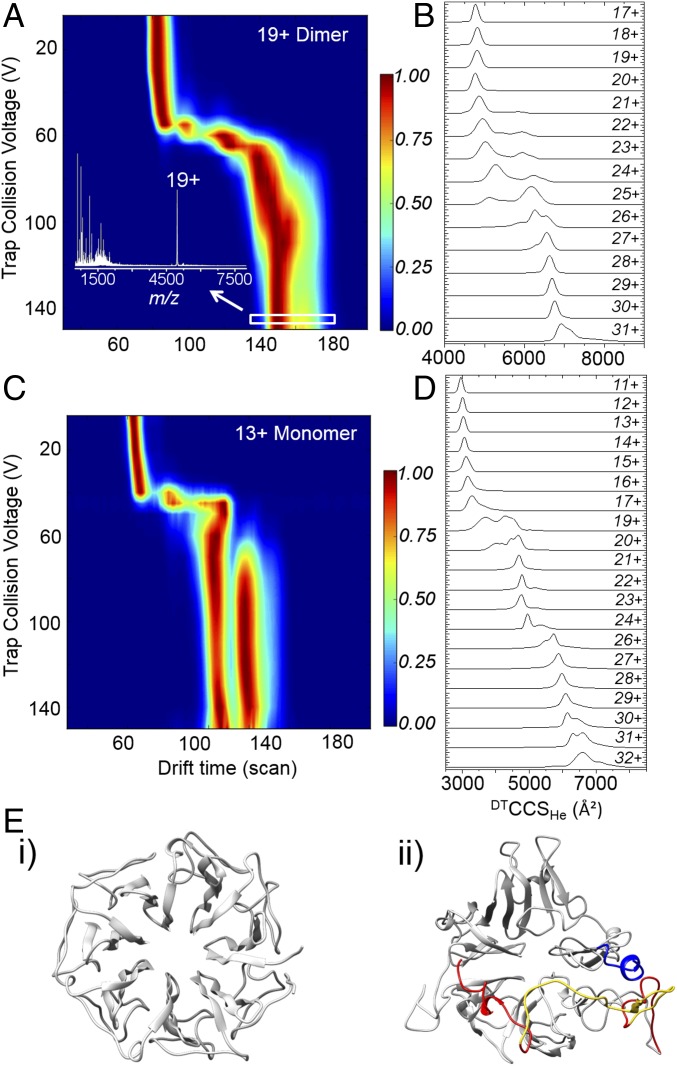
Collisional aIMS and simulated unfolding of full-length UVR8. All aIMS data were acquired using a modified Synapt G2-S mass spectrometer with traveling-wave ion mobility and ion mobility data were acquired using an Agilent 6560 ion mobility enabled Q-ToF mass spectrometer. (*A*) aIMS heat map of the compact 19+ charge state of full-length UVR8 dimer in the gas phase. The drift time of the 19+ charge state was measured as a function of trap collision voltage and converted to laboratory frame energy (charge × voltage). (*Inset*) Mass spectrum recorded at the highest activation energy. (*B*) ^DT^CCS_He_ of the full-length UVR8 dimer from ion mobility data. (*C*) aIMS heat map of the compact 13+ charge state of full-length UVR8 monomer in the gas phase. (*D*) ^DT^CCS_He_ of the full-length UVR8 monomer from ion mobility data. (*E*) Structures of the UVR8^12–381^ (*i* and *ii*) full-length UVR8 monomers (both top view) at the end of in silico unfolding simulations (*SI Appendix*, Fig. S13). The UVR8 core domains are colored gray, the N termini dark blue, the C termini red, and the C27 region yellow.

To test this hypothesis, we modeled in the C- and N-terminal tails on to the published (10) structure of UVR8^12–381^ (Fig. 1 and *SI Appendix*, Fig. S7) and ran MD simulations to calculate theoretical CCS values for different conformations of these tails. In the in silico model of the compact dimer, the theoretical CCS from MD simulations (*SI Appendix*, Fig. S3*C*) is ^Th^CCS_He_ = 5,391 ± 57 Å^2^ and a representative structure shows both C termini are close to the β-propeller core of the protein (Fig. 4 *E*, *i*). A typical simulated structure (*SI Appendix*, Fig. S3*D*) of the extended dimer, however, has a ^Th^CCS_He_ of 6,568 ± 94 Å^2^ and the C-terminal tails have unraveled into positions well away from the core domain with the C27 region required for binding to COP1 (6) now exposed (Fig. 4 *E*, *ii*). These data strongly suggest that the compact and extended forms of the full-length UVR8 dimer do indeed reflect different conformational states of the terminal tails, in particular the C terminus (which is substantially longer than the N terminus). It is worth noting at this stage that the purpose of our MD simulations was to explore whether the measured CCS values can be accounted for by different conformations of the C- and N-terminal tails, which in the dimer they can. They were not intended to thoroughly explore the conformational space accessible to the different forms of UVR8, but rather to provide candidate geometries to compare with experimental data. We therefore do not necessarily expect these precise geometries to be among the most significant populations found in nature. Consistent with our interpretation, lowering the concentration of the sample from 5 μM to ≤2.5 μM results in an increase in the proportion of extended species (*SI Appendix*, Fig. S8*A*), and some variation in the compact-to-extended ratio could also be observed upon changing instrumentation (*SI Appendix*, Fig. S9). It therefore appears that the different UVR8 dimer conformations can interconvert and that the equilibrium between the states is dependent on the protein environment. The ion mobility data of the full-length dimer, as presented in Fig. 5*B* and *SI Appendix*, Fig. S6*A*, also indicate that—rather than a continuum of conformations between the compact and extended forms as one might expect—a single conformational population dominates the extended form, with only a minor population representing a transitional state between it and the compact form.

Following exposure of the full-length UVR8 dimer to UV-B light in solution, the mass spectrum becomes dominated by the ion signal of the full-length monomer (Fig. 4*C*). A shorter illumination time (1 s) was chosen than for UVR8^12–381^ in Fig. 3 (10 s) to ensure that sufficient dimer remained for its mobility to be measured. The ion mobility data of this dimer were then compared with the nonirradiated dimer (*SI Appendix*, Fig. S10); no difference was evident. The monomer also presents in at least two distributions, with charge states ranging from z = 11+ to 14+ and z = 15+ to 32+. Experimental ^DT^CCS_He_ values from ion mobility data suggest the full-length monomer also adopts compact and extended conformations (Fig. 4*D* and *SI Appendix*, Fig. S6*B*). Much like the extended dimer, the z = 15+ to 32+ range for the full-length monomer comprises two subpopulations (with apexes at z = 19+ and z = 23+). Interestingly, the second population spans a very broad ^DT^CCS_He_ range (∼1,000 Å^2^), which suggests the monomer is highly dynamic and exists in a range of conformational states. Remarkably, this ^DT^CCS_He_ range for the full-length monomer is similar to that encompassing both the compact and extended forms of the dimer (Fig. 4*D*, dotted-dashed line), indicating this flexibility is in part facilitated by residues that are stabilized in the dimer. MD simulations of the full-length monomer in a compact form (Fig. 4 *E*, *iii*) resulted in a ^Th^CCS_He_ value of 3,292 ± 39 Å^2^ (*SI Appendix*, Fig. S3*E*), which is in the same region as those measured experimentally (Fig. 4*D*, green). By contrast, the ^Th^CCS_He_ value of the extended monomer (Fig. 4 *E*, *iv* and *SI Appendix*, Fig. S3*F*; 3,953 ± 63 Å^2^) falls right at the lower limit of the very broad range of experimental values (Fig. 4*D*, light blue). Even fully extending the C-terminal region in silico with the core constrained (*SI Appendix*, Fig. S11*C*) leads to a maximal ^Th^CCS_He_ value of 5,068 ± 126 Å^2^, which is still less than the ^DT^CCS_He_ range (∼5,600–7,000 Å^2^) of the main extended population.

We therefore hypothesized that as the monomer becomes more extended it does not simply represent an unfolding of the terminal tails but of the core protein fold as well. The beginning of this process is evident in the representative structure of the extended monomer following MD simulation in Fig. 4 *E*, *iv*. The core domain is unconstrained during these simulations and is clearly becoming less well-ordered than in the dimer structures (both UVR8^12–381^, Fig. 3*B*, *Inset* and full-length, Fig. 4 *E*, *i* and *ii*), the UVR8^12–381^ monomer structure (Fig. 3*F*, *Inset*), and the compact full-length monomer structure (Fig. 4 *E*, *iii*). It therefore appears that the homodimeric interface not only maintains the quaternary structure in the dark but also stabilizes the tertiary structure of the core domain in each full-length monomer. Because similar unfolding of the core domain is not evident in the monomer where the C and N termini have been truncated (i.e., the UVR8^12–381^ monomer; Fig. 3*F*, *Inset*), we hypothesized that the extended IDRs of full-length UVR8 serve to destabilize the β-propeller structure in the absence of the stabilizing cross-dimer interactions. Overall, we conclude from these data that the full-length UVR8 structure has access to conformations where one or more of the C and N termini are extended (Fig. 4 *E*, *ii* and *iv*) and even the core, β-propeller structure can adopt more extended states. The ion mobility data of the full-length monomer, as presented in Fig. 5*D* and *SI Appendix*, Fig. S6*B*, indicate that two conformational populations dominate the extended form, with a third, minor population representing a transitional state between them and the compact form.

### The UVR8 Dimer Is Highly Stable in the Dark, Whereas the Terminal Tails Are Conformationally Disordered.

It therefore seems likely that the activation of full-length UVR8 not only requires dimer dissociation triggered by light but also the sampling of conformational space to enable the C terminus to mediate protein–protein interactions and thus initiate signaling and regulate UVR8 function. Interestingly, our data indicate that the UVR8 dimer is a highly stable state. To investigate this further, collisional activation before ion mobility (aIMS) of full-length UVR8 was conducted in the absence of UV-B light (Fig. 5*A*). Here, the drift time of a mass-selected charge state is measured as a function of trap collision voltage (i.e., activation energy). It can be seen in Fig. 5*A* that the initial compact dimer structure of z = 19+ is stable up to an activation energy of ∼1,000 eV, after which there is a transition to longer drift times, which are consistent with a more extended dimer structure. A briefly stable, transitional distribution is evident at ∼1,300 eV which then unfolds further and remains stable up to 3,000 eV. The mass spectrum at this energy (Fig. 5*A*, *Inset*) reveals that whereas fragments corresponding to parts of the disordered tails are abundant in the spectrum no monomer is observed. These data follow a remarkably consistent pattern with the ^DT^CCS_He_ trend measured for the charge states of the full-length UVR8 dimer before photoactivation (Fig. 5*B* and *SI Appendix*, Fig. S5*A*): a compact, stable conformation and a transitional configuration leading to a second stable, extended state. The remarkable resilience of the dimer form in the absence of light was also shown by collision-induced dissociation (CID) (*Methods*), with the terminal tails being fragmented under collision while the dimer interface remained intact (*SI Appendix*, Fig. S12). Altogether, these data confirm the hypothesis supported by the computational data in Fig. 4: the compact and extended conformations of the full-length dimer correspond to different conformational folds of the terminal tails.

### The Disordered C Terminus Destabilizes the Core Structure in the Full-Length Monomer.

To test our hypothesis that the interactions at the homodimeric interface not only maintain the quaternary structure but also stabilize the β-propeller core of each monomer, we conducted aIMS of the compact, full-length monomer (Fig. 5*C*). The initial conformation is only stable up to an activation energy of ∼500 eV (compared with ∼1,000 eV for the compact dimer) and the subsequent transition is far more abrupt. Moreover, it transitions to drift times that are indicative of highly extended monomer conformations, consistent with the ion mobility data (Figs. 4*D* and 5*D* and *SI Appendix*, Fig. S6*B*). The aIMS data also suggest that there are two stable conformational families of highly extended monomer, whose drift times remain relatively constant as the laboratory frame energy is increased from ∼800 to ∼2,000 eV. This is supported by the native ion mobility data of the full-length monomer in Fig. 5*D*, where the two dominant populations of the extended monomer are present in the ions that present with z = 20+ to 24+ and 26+ to 30 +, respectively. In combination, the aIMS and ion mobility data of the full-length UVR8 monomer strongly suggest that, in the absence of the stabilizing cross-dimer interactions, the β-propeller fold of the core domain becomes destabilized when the terminal tails are extended.

It should be noted again that the core of the UVR8^12–381^ monomer, which lacks the IDRs, does not present with such conformational diversity of the core domain (Fig. 3). To test our previously stated hypothesis that the IDRs of UVR8, and in particular the longer C terminus, destabilize the tertiary structure of core domain in the full-length monomer, we conducted simulated unfolding of both the UVR8^12–381^ and full-length monomers (*SI Appendix*, Fig. S13). These data confirm that the core domain of the full-length UVR8 monomer is substantially less stable than when the terminal tails are absent (i.e., for the UVR8^12–381^ monomer). This is starkly illustrated by representative structures of the UVR8^12–381^ (Fig. 5 *E*, *i*) and full-length (Fig. 5 *E*, *ii*) monomers following these unfolding simulations. Whereas the UVR8^12–381^ monomer remains well-folded, with little deviation from the β-propeller fold of the published crystal structures (10, 11, 17) (Fig. 1*A*), the core domain of the full-length UVR8 monomer has become significantly disordered. From these data we therefore conclude that the extended IDRs act to convert the core fold from the β-propeller structure of the homodimeric state toward highly extended, stable conformations in the UVR8 monomer.

### The Extended UVR8 Dimer Dissociates into Monomers More Readily than the Compact Dimer.

As we have seen, the UVR8 dimer is a highly stable structure which is resistant to any dissociation into monomers when in the gas phase and in the absence of UV-B, even under unfolding conditions. Indeed, the UVR8 dimer still fails to form monomers in the gas phase under UV-B illumination (*SI Appendix*, Fig. S14). It is highly likely, therefore, that water molecules are required for the successful photoactivation of UVR8. The interfacial region of each monomer is highly charged and the dimeric structure is maintained in the dark by cross-dimer salt bridges (10, 11). A water molecule is thought to be ejected from the interfacial region following photoexcitation of the tryptophan chromophore; it has been proposed that this leads to disruption of a network of H-bonds and the cross-dimer salt bridges (17). For the dimers to convert to monomers following photoactivation rather than to immediately redimerize, our experiments indicate that additional water molecules that are absent in the gas phase must be required to enter the interfacial region and shield the complementary charges from one another.

**Fig. 6.**
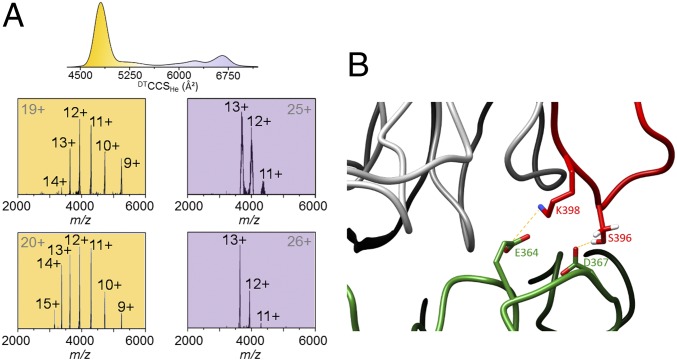
SID of the UVR8 dimer in the gas phase. (*A*, *Top*) Ion mobility data showing the compact (orange) and extended (purple) conformations of the full-length UVR8 dimer. (*A*, *Bottom*) The 19+ and 20+ charge states of the compact dimer each show an asymmetric dissociation pattern in the resulting monomer spectra (orange panels), whereas the 25+ and 26+ charge states of the extended dimer each show a symmetric dissociation pattern in the resulting monomer spectra (purple panels). (*B*) A representative structure of the compact full-length dimer conformation where the C- and N-terminal tails have been modeled on to the published (10) structure of the UVR8^12–381^ dimer. An example H-bonding pattern between the C terminus (red) of one monomer (the rest of which is colored gray) and the core domain of the opposite monomer (green) are illustrated. More possible patterns are shown in *SI Appendix*, Fig. S15.

Gas phase dissociation to monomers was only possible using surface-induced dissociation (SID) (Fig. 6 and *SI Appendix*, Fig. S16), which imparts significant amounts of energy onto the ion in a single, high-energy collision with a functionalized gold surface. A SID voltage of 100 V was sufficient to dissociate the dimer into monomeric units (Fig. 6*A*). SID of compact dimers (e.g., z = 19+ and 20+) results in an asymmetric dissociation pattern in the resulting monomer spectra (Fig. 6*A*, orange panels), with many charge states of the monomer evident. Extended dimers (e.g., z = 25+ and 26+), however, give a symmetric dissociation pattern in the resulting monomer spectra (Fig. 6*A*, purple panels) in which only a narrow range of charge states is produced. The broader charge-state distribution in the SID spectra resulting from the compact dimer state results from “scrambling” of charges along the interface. Such scrambling reflects the fact that more protons are transferred to one monomer of the dissociating dimer than to the other (hence the asymmetric charge distribution). Remarkably, what these data reveal is that the extended dimers dissociate into monomers much more readily than do the compact dimers, suggesting additional interactions between the monomers in the compact dimer. Indeed, MD simulations of the full-length dimer reveal a number of different H-bonding patterns are possible in the compact dimer between residues of the C-terminal tail that were missing in the published crystal structures (10, 11, 17) and residues on the core domain of the opposite monomer (Fig. 6*B* and *SI Appendix*, Fig. S15). Moreover, an asymmetric pattern suggests that there is a significant population of compact dimers where the number of interactions involving each C terminus is not equivalent, consistent with their predicted disorder (*SI Appendix*, Fig. S1). By contrast, the extended dimers dissociate in a far more facile manner, which leads to a lower number of charge states in the dissociated monomer (Fig. 6*A*, purple spectra).

## Discussion

The plant photoreceptor UVR8 has evolved to respond to and thus protect against the harmful effects of UV light (8) and was the first photoreceptor discovered to employ tryptophan residues from the protein backbone as photoactive chromophores (12). Crystal structures of the core domain (UVR8^12–381^) (10, 11) reveal that photoexcitation of a pyramid of four tryptophan residues disrupts a network of salt bridges that stabilizes the homodimeric structure, leading to dissociation into monomers. We have shown here that the evolution of the dimer and monomer signals of UVR8^12–381^ under UV-B illumination can be monitored using native ion mobility mass spectrometry, and that the decay of the former is correlated with the appearance of the latter. Furthermore, in the UVR8^12–381^ variant a single conformational family of dimers converts to a single conformational family of monomers (i.e., both are well-folded structures).

By contrast, we have revealed that full-length UVR8 possesses considerable conformational diversity, which is likely to be necessary for functional binding to signaling components. Full-length UVR8 and COP1 interact in the nucleus following UV-B illumination both *in planta* (15) and in protein extracts (12). Although UVR8^12–381^ can also monomerize, accumulate in the nucleus (6), and then interact with COP1 (16), it is missing a highly conserved 27-residue region of the C terminus (C27) that is required for the binding to be functional. We show that the full-length UVR8 dimer exists in two main conformational families, one compact and one extended, with a transitional state between them. The compact and extended forms of the dimer are defined by different conformational states of the intrinsically disordered terminal tails, in particular the longer C terminus, and the extended form is far more conformationally diverse. Access to extended conformations is consistent with published size-exclusion chromatography, where the full-length dimer elutes at an apparent molecular mass of ∼150 kDa (11) (cf. the sequence mass of ∼100 kDa), and with small-angle X-ray scattering analysis (10). The fact that we observe two main populations of compact and extended forms and not a continuum of states suggests there is at least an element of order to the C-terminal flexibility. Consistent with this, our MD simulations of the compact dimer reveal these conformations are likely to be stabilized by interactions between the C terminus of one monomer and the core domain of the opposite monomer. Moreover, previous modeling of the C terminus suggested that residues 400–421 and 432–440 are likely to be α-helical, with the remaining, unfolded residues predicted to be flexible (19).

In the absence of UV illumination the UVR8 homodimeric structure is very stable. Indeed, we show that the dimer remains intact following CID, despite the C- and N-terminal tails undergoing fragmentation. This is consistent with that fact that unboiled samples of the isolated protein in solution remain dimeric in the dark even under the denaturing conditions of SDS/PAGE (10, 11). Remarkably, we further reveal that in the gas phase even UV-B illumination is insufficient to cause significant dimer dissociation. This observation suggests that photoconversion to monomers is likely to proceed in two stages. First, photoexcitation in solution triggers disruption of the cross-dimer salt bridges, causing initial separation of the monomers. Second, water molecules surrounding the homodimer and within the central, water-filled tunnel (10) are required to enter the interfacial space to shield the salt bridge partners from one another and thus enable the monomers to separate completely. These water molecules will mostly be absent in the gas phase and are therefore not available in sufficient numbers to stop the salt bridges from reforming. We found that SID provided sufficient energy to cause the dimer to dissociate into monomers in the gas phase. SID is equivalent to a substantial increase in temperature, which is consistent with boiling of unilluminated aqueous samples of dimer being necessary to produce monomers on SDS/PAGE (10, 11). SID also reveals that the extended dimer converts to monomers more readily than the compact dimers do. This may well be owing to the additional interactions evident between the C terminus of one monomer and the core domain of the other monomer in the compact form (Fig. 6*B* and *SI Appendix*, Fig. S15). The extended dimer conformation could therefore represent a state “primed” for activation, leading to a monomeric form with an extended C terminus that is ready for functional interaction with COP1.

The UVR8/COP1 interaction is UV-B–dependent (15), and the dimer with an extended C terminus is therefore not sufficient to facilitate this interaction. Based on this, both dissociation into monomers and access to the extended conformations of the C terminus that we observe are the minimum requirement for functional binding and therefore signal transduction. Much like for the full-length dimer, and in contrast to the UVR8^12–381^ monomer, the native mass spectrum and ion mobility data for the full-length monomer reveal numerous conformational states. These are consistent with a single compact form, a transitional state, and two highly extended, but nevertheless stable, states. In contrast to the full-length dimer, the extended monomeric states cannot be accounted for by extended conformations of the C and N termini alone, and also require unfolding of the β-propeller structure of the core domain. Extended monomer conformations are consistent with published in vivo data; an additional band appears above the presumably compact monomer on SDS/PAGE of unboiled protein extracts from *Arabidopsis uvr8-1–*expressing GFP-UVR8 variants (20). This band increases in intensity with UV-B illumination of GFP-UVR8, is present but unresponsive to UV-B in the constitutive monomer GFP-UVR8^R286A^, and is apparently not detected for the constitutive dimer GFP-UVR8^W285F^. All of these trends are consistent with our data. Although one might expect that the adoption of such highly extended conformations would make the UVR8 monomer more susceptible to degradation in vivo, this seems not to be the case (21).

Highly extended monomeric states suggest that the cross-dimer salt bridges not only serve to stabilize the homodimeric structure in full-length UVR8 but also the β-propeller structure of the core domain. We show that the presence of the terminal tails, and in particular the longer C terminus, destabilizes the core fold in the absence of the cross-dimer interactions. It has been previously demonstrated that COP1 interacts not only with the C terminus of UVR8 but also with the UVR8 core domain (16). We therefore propose that in order for the rather large COP1 protein (658 residues) (22) to successfully interact with the UVR8 core domain the latter must unfold to a highly extended conformation. To achieve full, functional binding with COP1, it appears that the intrinsically disordered C terminus not only interacts directly with COP1 but also serves as an “intramolecular allosteric effector” (23) that enables the core domain to form additional, stabilizing protein–protein interactions. Such a role in allosteric control is analogous to those reported recently for IDRs in the human glucocorticoid receptor (GR) transcription factor (23) and similar systems where allosteric mechanisms reflect changes to the ensemble-average structure of proteins (24). Indeed, similar to GR, the full-length UVR8 homodimer appears to be in a state of “energetic frustration” (23) where the destabilizing effect of the C terminus on the core domain is negated by the stabilizing effect of the cross-dimer interactions. The balance of these opposing forces is then tipped toward the destabilizing effect of the C terminus IDR upon light-activated dimer dissociation.

It is possible that light plays a dual role in this regard. Dynamic crystallography of UVR8^12–381^ reported partial unwinding of the β-propeller fold in response to UV-B (17), which appears to be driven by motions in blades 5 and 6 (Fig. 1*A*), which contain the key residues of the chromophore, W285 and W233, respectively. In combination with our data, this suggests that structural rearrangement of the core domain is triggered by light not only to effect dimer dissociation but also to actively trigger destabilization of the core fold. In support of this proposal are data from the W285A variant, which is a photoinactive constitutive monomer. It interacts functionally with COP1 in vivo (25), but this interaction is not as strong as that of WT UVR8 (12) because it only does so via its C terminus (16). This is consistent with one role of photoactivation’s being to trigger the unwinding of the β-propeller core. Further, one cannot rule out the possibility that full activation of UVR8 is a two-photon process, with the second photon absorbed by the monomers to effect a more complete unfolding of the core structure. Consistent with this notion, experiments with a monomeric but photoactive double mutant of UVR8 (D96N/D107N) show that the monomer is able to absorb UV-B and initiate responses in vivo, indicating that dimer photoreception is not essential for UVR8 function (26).

**Fig. 7.**
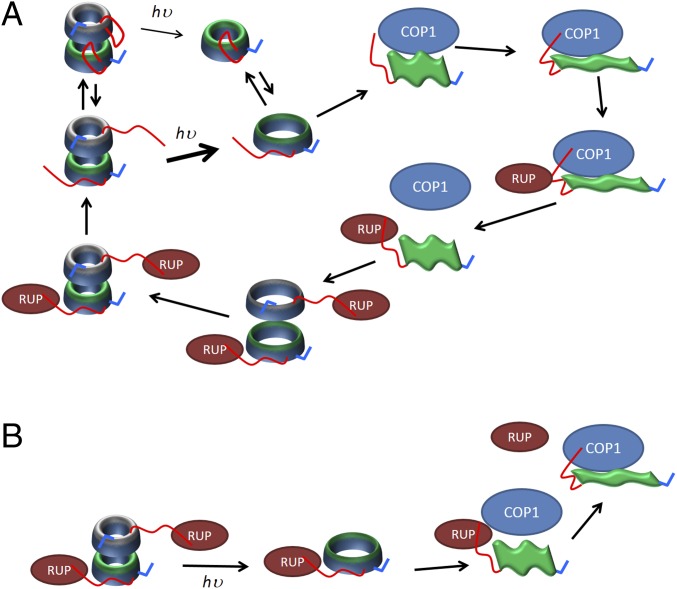
Predictive mechanisms of the protein interactions that mediate UVR8 signaling and regulation. For a full discussion of each mechanism refer to the main text. Consistent with the color scheme used throughout this article, the full-length UVR8 homodimer is depicted using one green and one gray core domain for the two monomers, each with a red C-terminal and blue N-terminal tail. *hυ*, absorption of a photon. (*A*) Cartoon illustrating the proposed mechanism for photodissociation of the compact and extended UVR8 dimers into the various monomer conformations and subsequent interactions between the extended UVR8 monomers (green flag-like structures) and COP1/RUP proteins. (*B*) Possible mechanism for displacement of RUP by COP1 should RUP remain bound to UVR8 during photoactivation.

We thus propose a predictive mechanism in Fig. 7*A* that is consistent with our data and the discussion above. The compact and extended UVR8 dimers represent different conformations of the disordered C- and N-terminal tails and are in equilibrium. Photoexcitation of the tryptophan chromophore disrupts the cross-dimer salt bridges and triggers both dimer dissociation and unwinding of the core domain. Dissociation occurs preferentially from the extended dimer and requires water molecules to enter the intermonomer space to shield salt bridge partners from one another. The C terminus in the resulting extended monomers is thus both available for functional interaction with COP1 and serves to further destabilize the core domain to yield highly extended conformations. A two-step mechanism has previously been proposed for subsequent interaction between the UVR8 monomer and COP1 (16): a stabilizing interaction between COP1 and the UVR8 core domain followed by interaction between COP1 and UVR8 C terminus. Here we propose that for the initial stabilizing interaction to take hold the UVR8 monomer must adopt a highly extended conformation. The entropic loss on forming this interaction from a more extended state may be enthalpically compensated by a very specific interaction with COP1, or may lead to so-called fuzzy interactions (27) at several sites on the receptor.

The UVR8/COP1 complex up-regulates RUP proteins (28), which are negative regulators of UVR8 that only interact with the UVR8 C terminus and do so in both its monomeric and dimeric state (6, 29). Presumably RUP needs to displace COP1 to repress UVR8 function, which they could achieve through competitive binding to the C terminus (Fig. 7*A*). The RUP proteins could then help bring the UVR8 monomers back together while mitigating the destabilizing influence of the C terminus and enabling the cross-dimer interactions to promote refolding of the β-propeller core. The question has also been asked about whether it is necessary for the RUP proteins to disassociate from UVR8 following reassembly of the homodimer and during subsequent rounds of photoactivation (16). Fig. 7*B* offers one possible mechanism whereby docking of COP1 to the extended core domain might facilitate displacement of the RUP proteins should they indeed remain bound.

## Conclusions and Outlook

Organisms throughout nature respond to their ambient light environment through the action of photoreceptor proteins, with functions as various as shade avoidance by plants, neuronal gating, and circadian photoentrainment. In most cases, absorption of light results in structural changes in the protein that enable interaction with signaling partners. Such interactions are often mediated by disordered regions. We have demonstrated the power of native mass spectrometry to elucidate the conformational diversity of the plant UV photoreceptor UVR8. In addition to monitoring the well-described light-triggered changes to quaternary structure, this approach enabled us to detail the different conformations of the flexible N- and C-terminal tails and how these IDRs influence the structure of the core domain in preparation for signaling. These data also serve as a good example not only of how IDRs are important for signaling interactions but also of how the conformation of ostensibly well-folded, highly stable domains can and do change according to functional requirements. Over recent years, native ion mobility mass spectrometry has emerged as a powerful technique with which to “capture the repertoire of conformational states adopted by protein assemblies” (30) from a wide variety of biological systems. The experimental approach described here makes use of a simple illumination setup that can be adapted straightforwardly to investigate the structural dynamics of any photoreceptor protein. Moreover, the versatility of photoreceptor function means that they are commonly reengineered as optogenetic tools to enable spatiotemporal control over target processes in biology using light. The approach employed here should also prove a powerful addition to the range of techniques with which to investigate, understand, and optimize the structural dynamics important to optogenetic function.

## Methods

### Protein Expression and Purification.

The genes that encode full-length UVR8 from *Arabidopsis thaliana* (residues 1–440) and the variant where the C and N termini are truncated (leaving residues 12–381, UVR8^12–381^) were cloned into a modified pET28a (Novagen) expression vector using NcoI and NotI, to provide N-terminal 7xHis and Strep II affinity tags, and expressed as SUMO fusion proteins as described previously (10). Briefly, the plasmids were transformed into *Escherichia coli* Rosetta 2 (DE3) pLysS cells (Novagen) and a single colony inoculated into LB media. Cells were grown overnight at 37 °C (preinoculum). Terrific Broth was then inoculated with the preinoculum, and cells were grown to an OD_600_ ∼ 1.0 and induced with 60 µM isopropyl-beta-d-thiogalactopyranoside at 16 °C for 20 h. Cells were harvested by centrifugation, snap-frozen in liquid nitrogen, and stored at −80 °C. Cells were thawed in buffer A (100 mM Tris⋅HCl, pH 8.0, 500 mM NaCl, 20 mM imidazole, 1 mM β- mercaptoethanol, 10% glycerol, and protease inhibitors) and lysed by incubation with BugBuster reagent for 1 h at room temperature and the cell debris was removed by centrifugation. The supernatant was collected, filtered through a 0.45-µm membrane, incubated in batch with 5 mL Ni-NTA SuperFlow (Qiagen) for 1 h, washed in buffer A, then buffer B (100 mM Tris⋅HCl, pH 8.0, 500 mM NaCl, 10 mM ATP, 2.5 mM MgCl2, 1 mM β-mercaptoethanol, and protease inhibitors), and finally with buffer C (100 mM Tris⋅HCl, pH 8.0, 150 mM NaCl, 1 mM β-mercaptoethanol, and protease inhibitors). Protein was eluted with buffer C supplemented with 300 mM imidazole. Eluted protein was incubated in batch with 1 mL Strep-Tactin XT Superflow (IBA Lifesciences) for 1 h and washed with buffer C. UVR8 was cleaved and eluted from the resin by incubation with SUMO protease at 4 °C for 16 h, and concentrated to 10 mg/mL using a Vivaspin 2 (10,000 molecular weight cutoff; Sartorius). UVR8 was then dialyzed to 250 mM ammonium acetate, pH 6.8, using a Slide-A-Lyzer Dialysis Cassette (10,000 molecular weight cutoff; ThermoFisher Scientific). SDS/PAGE of both purified variants is shown in *SI Appendix*, Fig. S17.

### Mass Spectrometry and Irradiation Experiments.

Mass spectrometry experiments were performed on a modified traveling-wave ion mobility-enabled Synapt G2-S (Waters) described previously (31), an Ultima Global (Micromass) optimized for high mass range, an Agilent 6560 Ion Mobility Q-TOF (Agilent), and a modified Synapt G2 with a linear drift tube in place of the standard triwave assembly. nESI tips were pulled in house from borosilicate glass capillaries on a Flaming/Brown Micropipette Puller (P-97; Sutter Instrument Co.). The sources of the Synapt and Ultima instrument were modified to accommodate a high-powered 280-nm LED (Thorlabs) focused on the nESI tip (Fig. 2), allowing irradiation of the sample solution before it enters the mass spectrometer; 5–20 μM UVR8 full-length and core were measured before, during, and after irradiation. Data were analyzed using MassLynx v4.1 (Waters Corporation), OriginPro 9.1 (OriginLab Corporation), and Microsoft Excel 2010 (Microsoft).

### Native Mass Spectrometry and Ion Mobility Mass Spectrometry CCS Measurements.

Native mass spectra were recorded on an Ultima Global (Micromass) employing gentle source conditions with a capillary voltage of ∼1.4 kV, cone voltage of 35 V, and all radio frequencies set to 0. CCS measurements were performed on an in-house-modified Synapt G2 mass spectrometer. The original traveling-wave ion mobility assembly of the instrument was replaced with a linear drift tube with a length of 25.05 cm. Measurements were made in helium at 298.15 K and a pressure of 1.98–2.01 Torr was kept constant throughout each run. Spraying conditions were set to be as gentle as possible with an applied capillary voltage ranging from 1.1 to 1.3 kV, a sample cone voltage of 15 V, and source temperature of 298.15 K. Drift times were recorded at a minimum of six voltages ranging from 220 to 120 V. All experiments were conducted in triplicate over at least two separate days. The drift times obtained were converted into rotationally averaged ^DT^CCS_He_ values using the Mason–Schamp equation (32).

Additional CCS measurements were performed in nitrogen on an Agilent 6560 Ion Mobility Q-TOF. VCap was set to 1.8 kV with a fragmentor voltage of 500 V. The drying gas was down-regulated manually to 250 μL⋅min^−1^ and drift times were recorded over five voltages ranging from 1,700 to 900 V. Measurements were made in triplicate over at least two separate days. Drift times were converted into ^DT^CCS_N2_ using the Mason–Schamp equation. ^DT^CCS_N2_ obtained were further converted into a single, global ^DT^CCS_N2_ for each species by summing the ^DT^CCS_N2_ of all measured charge states weighted according to their individual intensity in the mass spectrum.

### CID and Collisional aIMS.

To follow aIMS, the species of interest was mass selected in the quadrupole of a Synapt G2-S and its drift time measured as a function of trap collision voltage which was increased incrementally from 4 to 150 V. Fragmentation at a selected collision voltage (110–130 V) was analyzed and compared between species. Heat maps were plotted using CIUsuite (33).

### Gas Phase Illumination.

The gas phase illumination experiments were conducted using a modified TWIMS-enabled Q-ToF mass spectrometer that allows UV photodissociation (UVPD) within the instrument, by overlapping of a laser beam with the ion beam within the transfer cell region (31). The instrument modification includes an ion gating and trapping procedure that allows ions to be stored for several seconds, enabling UVPD. Illumination was achieved from the fourth harmonic (266 nm) of Continuum Minilite II Nd:YAG laser at a repetition rate of 10 Hz. Typical times used in these experiments were 1-s filling and 1-s trapping. During these periods, the laser was firing and ions experienced between 10 and 20 laser shots. The energies used range between 0.4 and 1 mJ per pulse. The overall transmission of the energy between the output of the laser and the entrance of the transfer cell was measured as 50%. As a control, equivalent experiments were conducted in the solution phase by placing the sample solution in the path of the 266-nm laser beam outside of the mass spectrometer. This was to confirm that UVR8 dissociates into monomers under similar conditions but in the solution phase (*SI Appendix*, Fig. S14*D*).

### SID.

The trap cell of the Synapt G2-S instrument was replaced with a shortened version and a SID device inserted between the shortened trap cell and ion mobility cell. Voltages were optimized to maximize transmission through the device. The species of interest were mass-selected in the quadrupole and directed toward a gold surface where they experience a single high-energy collision. Fragments were then refocused and guided through the rest of the instrument for analysis.

### Constructing an Initial Model of Full-Length UVR8.

I-TASSER (34–37) was used to generate models of the N- and C-terminal loops missing from the crystal structure. First, models for each of the two full-length UVR8 monomers were generated in I-TASSER from the full-length UVR8 sequence using the truncated crystal structure (Protein Data Bank ID code 4dnw) as a template. The models were then aligned with the crystal structures, and the coordinates of the modeled N- and C-terminal loops were grafted onto the original truncated structure. Using the loops from the top-scoring I-TASSER models (as ranked by I-TASSER’s C-score) for each chain leads to clashes between the C-terminal loops since these loops were built extending into the dimer interface (*SI Appendix*, Fig. S7 *A* and *B*). Therefore, the model with the second-highest score for each was used (*SI Appendix*, Fig. S7*C*). Of course, there is significant uncertainty as to the actual conformations of the terminal loops. The purpose of the modeling carried out in this study, however, is not to generate definitive conformations of the compact and extended forms of the protein, which would require far more extensive sampling than carried out here, but rather to demonstrate the effect of changes in loop conformations on CCS values by generating reasonable examples of the compact and extended forms. Crucially, for both the full-length monomer and dimer two methods were used to generate an extended form—using solvated and gas phase dynamics from different starting points—which resulted in similar ^Th^CCS_He_ values (*SI Appendix*, Figs. S3 and S11).

### MD Simulations of Solvated Protein.

MD simulations of the solvated protein were run using single-precision Gromacs 5.0.4 (38) with the Amber 14 force field (39). All simulations used a 10-Å cutoff for electrostatic and van der Waals interactions, the lincs algorithm (40) for bond constraints, a 2-fs time step, particle mesh Ewald electrostatics, and periodic boundary conditions. The system setup was as follows: (*i*) The protein was placed in a solvation box of minimum 12 Å around the protein and counter ions were added and (*ii*) energy minimization was carried out and the system was thermalized at 300 K for 100 ps with constant pressure and 100 ps with pressure coupling (using the Parrinello–Rahman barostat), with constraints on the protein. All subsequent MD simulations in solvent were carried out using pressure coupling. First, 50 ns of conventional MD simulations were run, followed by 20 ns of simulated annealing to encourage additional conformational sampling, with the following 1.25-ns cycles: 1 ns at 300 K, 50 ps of heating to 350 K and 150 ps at 350 K, and 50 ps of cooling to 300 K. To analyze the hydrogen bonding patterns between UVR8 monomers (Fig. 6*B* and *SI Appendix*, Fig. S15), we ran additional MD simulations starting from the structure at the end of 20 ns of annealing. Running simulations with different starting velocities allows for greater sampling, so we ran two 80-ns simulations, one using the velocities from the end of the annealing simulation and the other randomized velocities, as shown in shown in *SI Appendix*, Fig. S15.

### Calculating the Theoretical CCS (^Th^CCS_He_).

To obtain structures for CCS calculations, gas phase simulations were run at 310 K. These were run using double-precision Gromacs 4.6.1 (38) using twin range cut-offs for electrostatic interactions, infinite coulombic and van der Waals cut-offs (technically 999.0 nm), and a 2-fs time step. The setup consisted of energy minimization followed by 50 ps of thermalization at 250, 280, 290, and 300 K. For the UVR8^12–381^ monomer and dimer, the crystal structure (Protein Data Bank ID code 4dnw) was used as the starting point. For the full-length monomer and dimer, the starting point was the I-TASSER model after the 20-ns simulated annealing described in *Constructing an Initial Model of Full-Length UVR8*. CCS values were calculated using EHSSrot (41). Every 500 ps, the ^Th^CCS_He_ values are the averages from the last 10 ns of these simulations, and the representative structures shown in Fig. 4 are those whose ^Th^CCS_He_ values are closest to this average.

### Generating Extended Conformations.

An initial extended conformation of the dimeric protein was created by pulling the center of mass of the α-carbons of the C terminus (residues 378–440) from the center of mass of the α-carbons of the core of each monomer at a rate of 0.01 Å/ns for 10 ns (*SI Appendix*, Fig. S7*D*). This structure was then relaxed for 20 ns using the same annealing procedure as described above for the compact dimer, which was sufficient for convergence of the radius of gyration (*SI Appendix*, Fig. S7*E*). The resulting structure was taken as a starting point for generating gas phase structures for calculating CCS values for the dimer as well as the monomer. To test the effect of maximally extending the C-terminal region of the dimer and monomer, the C terminus was pulled during gas phase simulations (since the water box would be prohibitively large for the fully extended state) at a rate of 0.02 Å/ns for 10 ns (*SI Appendix*, Fig. S11 *A* and *C*). Here, position constraints were applied to the core structure, as otherwise partial unfolding of the protein was observed (presumably, unfolding the C terminus in the gas phase requires significantly more energy than in solution). The protein was then allowed to relax with unrestrained MD simulations (*SI Appendix*, Fig. S11 *B* and *D*) to generate additional extended conformations.

### Unfolding Simulations.

Unfolding simulations were performed to qualitatively assess the relative stability of the UVR8^12–381^ and extended, full-length monomers, starting from the structures and velocities after 10 ns of 300-K MD. Simulated annealing simulations were performed with the temperature cycled between T_1_ and T_2_ with a periodicity of 1 ns: 400 ps at T_1_, 100 ps heating to T_2_, 400 ps at T_2_, and 100 ps cooling to T_1_ with the following T_1_–T_2_ values: 300–400 K, 350–425 K, 350–450 K, and 350–500 K. The rmsd for the UVR8^12–381^ residues for each structure (*SI Appendix*, Fig. S13) suggests that the full-length monomer is significantly less stable, as the rmsd for the core monomer does not increase significantly even after 10 ns of the harshest annealing. Fig. 5*E* shows each structure at the end of this annealing simulation.

## Supplementary Material

Supplementary File
