# Supplementary material for: Native mass spectrometry reveals the conformational diversity of the UVR8 photoreceptor
Source: Proc Natl Acad Sci U S A. 2019 Jan 4;116(4):1116–25. doi: 10.1073/pnas.1813254116 (PMC6347689; doi:10.1073/pnas.1813254116)
Supplement: Supplementary File [file pnas.1813254116.sapp.pdf]

Native Mass Spectrometry Reveals the Conformational Diversity of the UVR8 Photoreceptor

Inês S. Camacho,[1,2,3,4] Alina Theisen, [1,2,3] Linus O. Johannissen,[1,3] L. Aranzazú Díaz-Ramos,[5] John M. Christie,[5] Gareth I. Jenkins,[5] Bruno Bellina,*[1,2,3] Perdita Barran,*[1,2,3] and Alex R. Jones*[1,2,3,4]

1. *School of Chemistry, The University of Manchester, Manchester, M13 9PL, UK;*
2. *Photon Science Institute, The University of Manchester, Alan Turing Building, Oxford Road, Manchester, M13 9PL, UK;*
3. *Manchester Institute of Biotechnology, The University of Manchester, 131 Princess Street, Manchester, M1 7DN, UK;*
4. *National Physical Laboratory, Hampton Road, Teddington, Middlesex, TW11 0LW, UK;*
5. *Institute of Molecular, Cell and Systems Biology, College of Medical, Veterinary and Life Sciences, Bower Building, University of Glasgow, Glasgow, G12 8QQ, UK*

www.pnas.org/cgi/doi/10.1073/pnas.1813254115

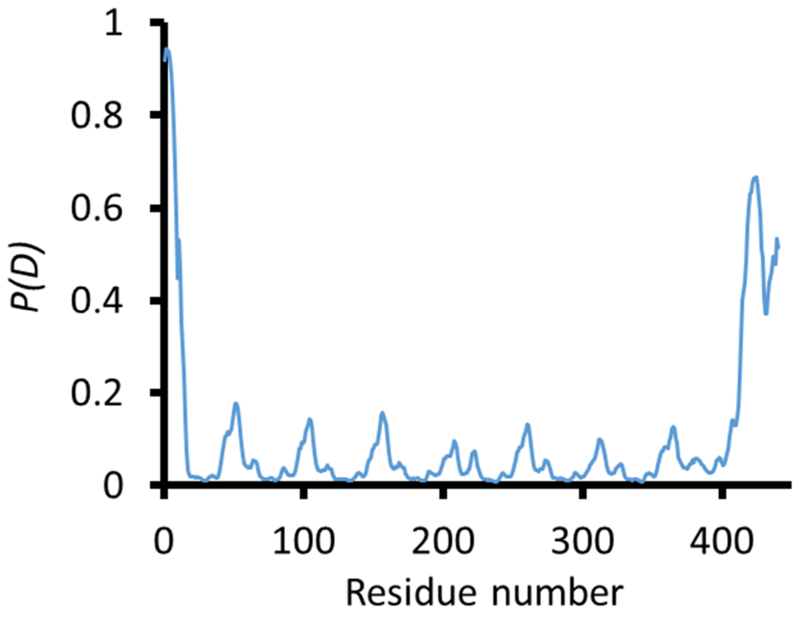

Figure S1. Disorder prediction for full-length UVR8 using the SPOT-D tool (1). A $P(D) > 0.5$ is considered a disordered region. Both the N- and C- terminal tails of UVR8 are therefore predicted to be highly disordered.

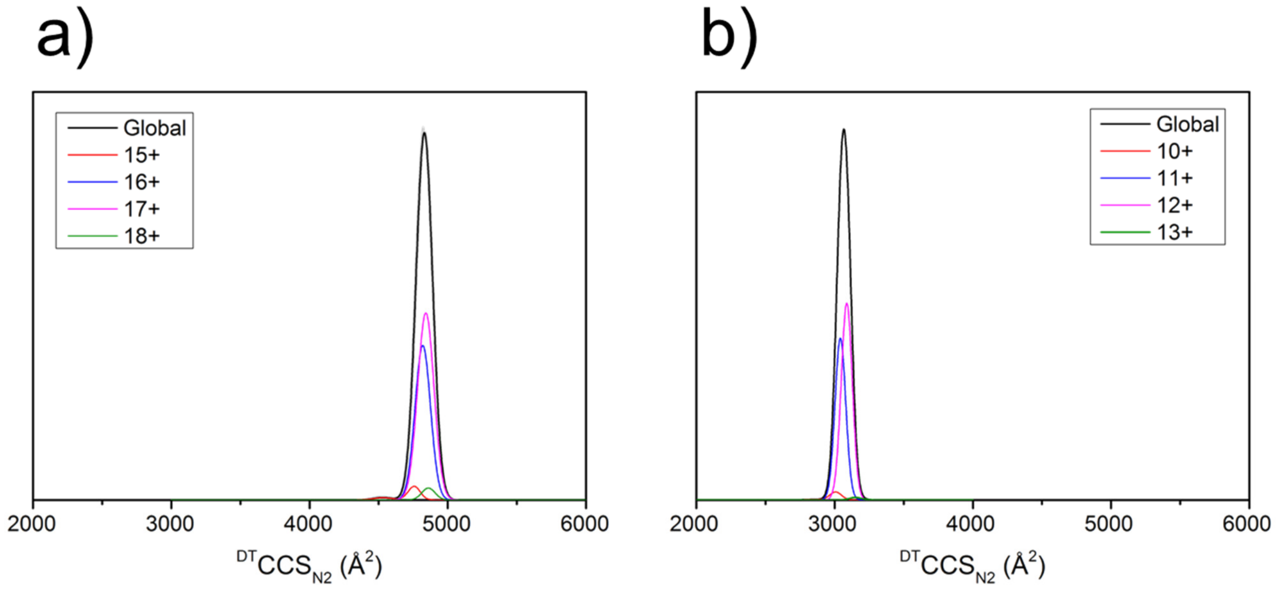

Figure S2. Collision cross section distributions, $^{DT}CCS_{N2}$, of 5 μM UVR8$^{12-381}$ dimer (**a**) and monomer (**b**) in 250 mM ammonium acetate measured in nitrogen using an Agilent 6560 ion mobility enabled Q-ToF. Equivalent $^{DT}CCS_{He}$ of the UVR8$^{12-381}$ dimer and monomer measured in helium on a Synapt G2 modified with a linear drift tube are in Figures 3b) and f), respectively.

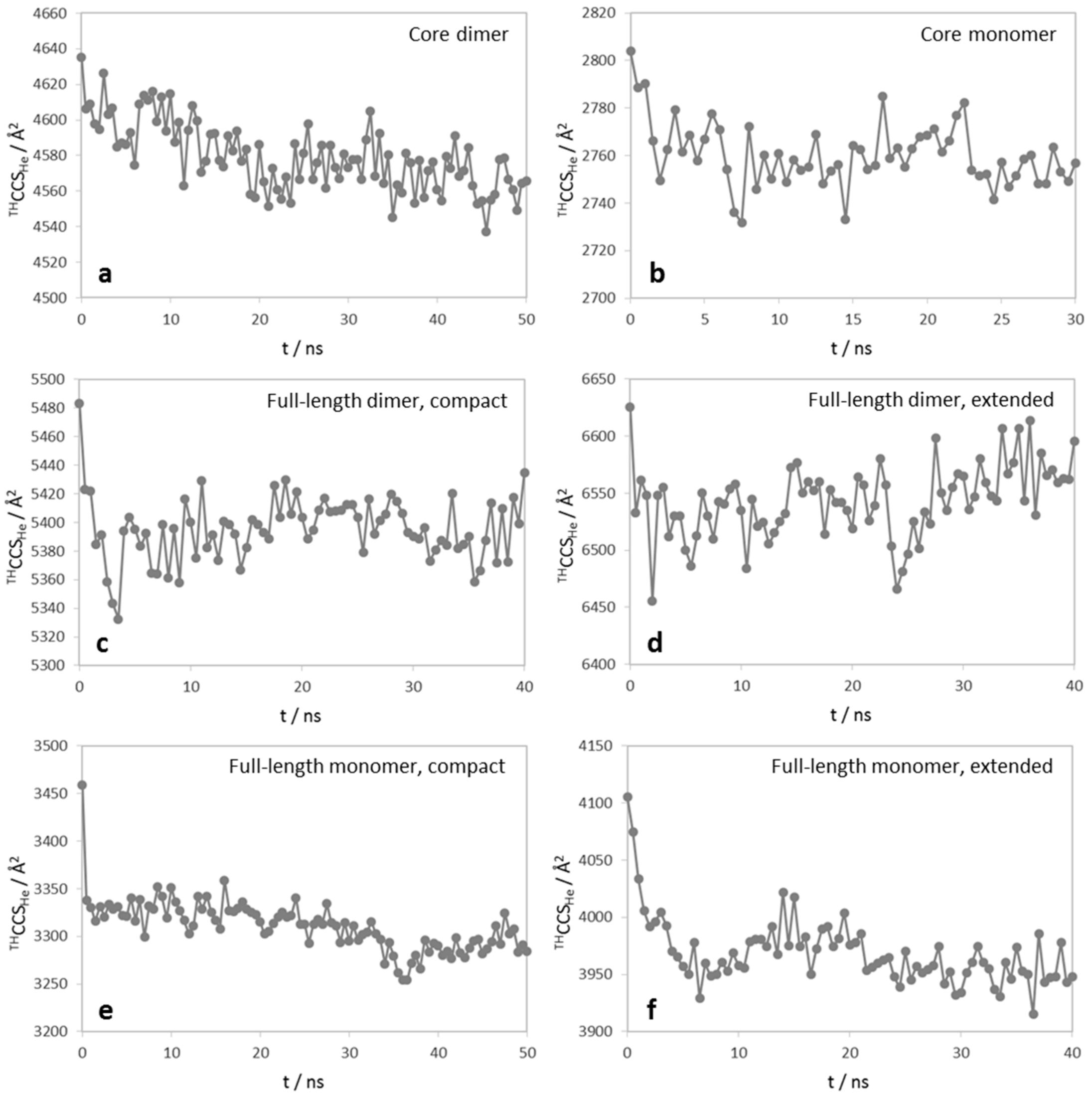

Figure S3. Computed, theoretical collision cross section ($^{Th}CCS_{He}$) values *vs* time for gas-phase MD simulations of the energy minimized structures of the UVR8 core domain (UVR8$^{12\text{-}381}$) (**a**-**b**) and full-length UVR8 (**c**-**f**). The structures of UVR8$^{12\text{-}381}$ are published (2), and for the full-length structures we have modelled in the C- and N-terminal tails (see Supporting Figure S7 and Methods section for details). The $^{Th}CSS_{He}$ values quoted in the main text are the averages for the last 10 ns of each simulation. Each simulation was halted when the $^{Th}CCS_{He}$ value had stabilized for at least 10 ns.

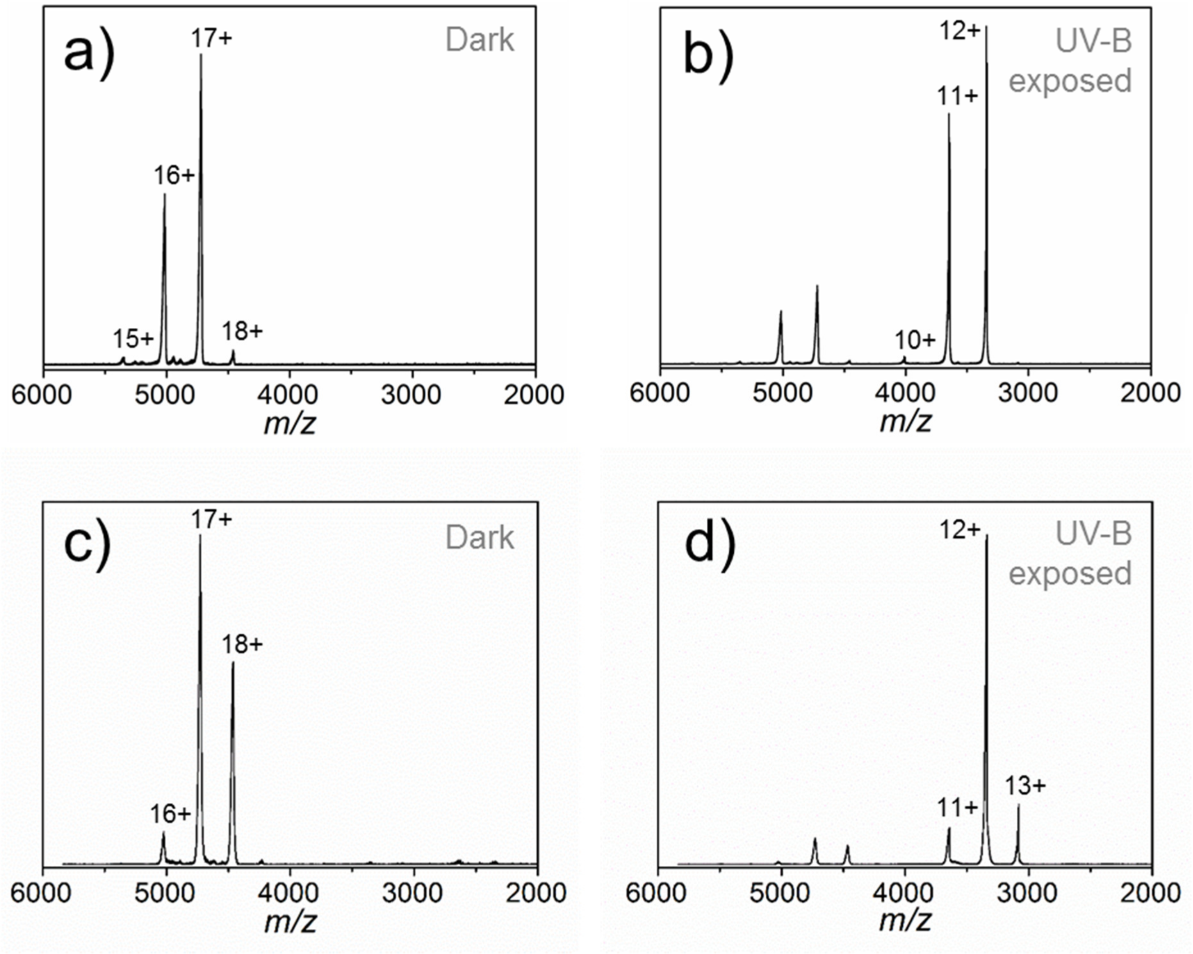

Figure S4. Native mass spectra of the UVR8$^{12\text{-}381}$ dimer (**a&c**) and monomer (**b&d**) measured using an Agilent 6560 Ion Mobility Q-ToF (**a&b**) and a Synapt G2 (**c&d**). Both show comparable data to those observed in the Ultima Global (Figure 3).

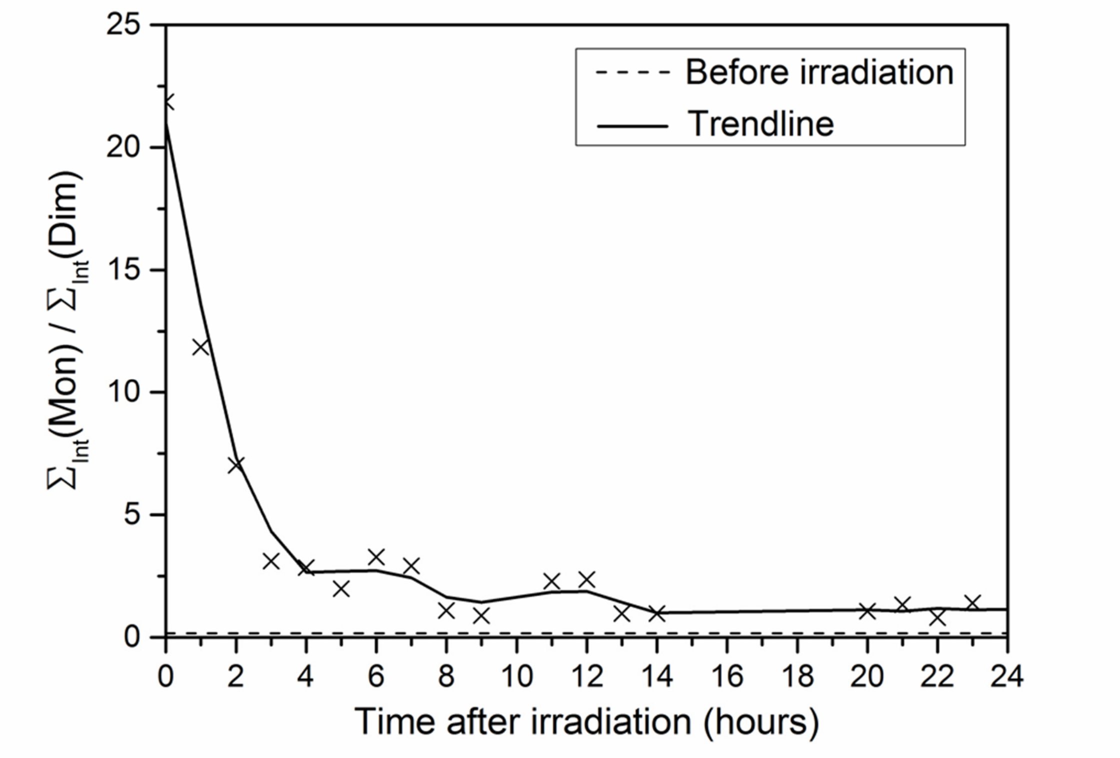

Figure S5. Monomer:dimer ratio calculated from the combined signal intensities (Σ_{int}) as a function of time following 10 s of UV-B illumination. Significant redimerisation of UVR8$^{12\text{-}381}$ occurs within 12 hours and is complete within 24 hours. Although on a similar order of magnitude, this appears to be slightly faster than measured previously by SDS-PAGE, where such dark reversion was only complete after 48 hours (2). Two factors are likely to explain this apparent disparity. First, redimerisation of UVR8 monomers is a bimolecular process and will therefore follow second order kinetics, which are highly dependent on sample concentration. The concentration in the ion source (5 μM) is likely to be higher than that used for the SDS-PAGE samples. Second, the tip surface-area to sample volume ratio within the ion source is high, and therefore nucleation sites for redimerisation are likely to be numerous.

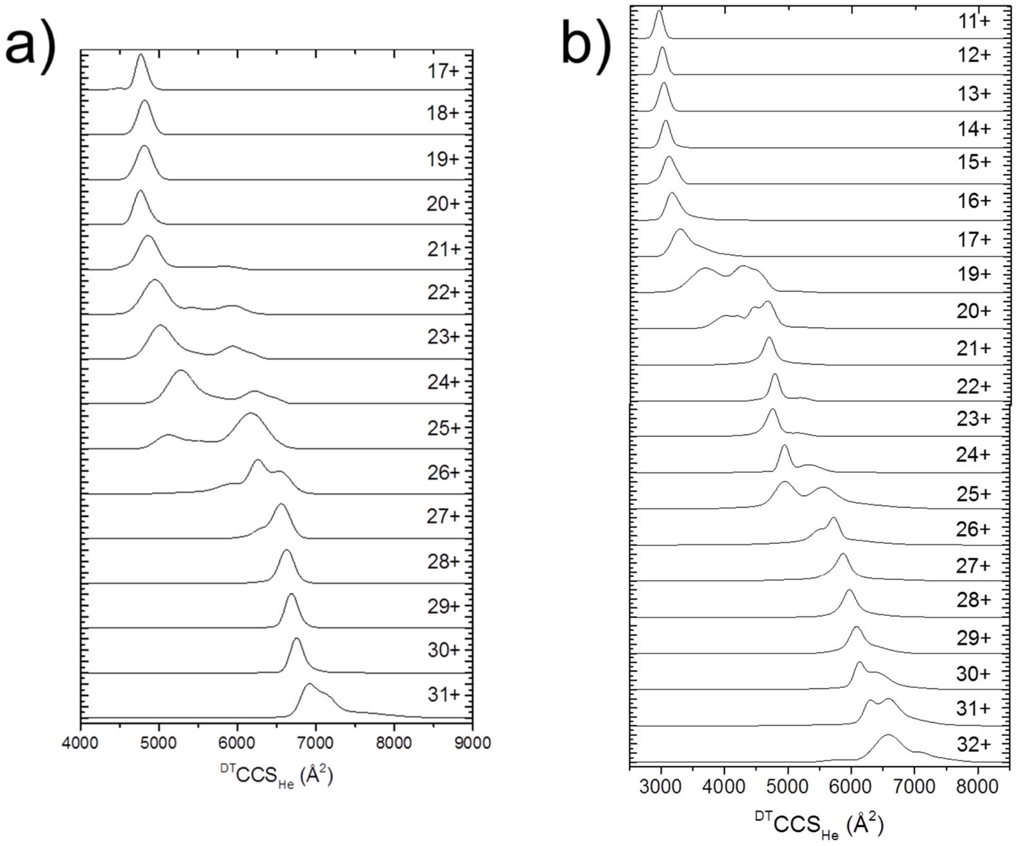

Figure S6. $^{DT}CCS_{He}$ of full-length UVR8 measured on an Agilent 6560 ion mobility enabled Q-ToF mass spectrometer. **a)** $^{DT}CCS_{He}$ of full-length UVR8 dimer. **b)** $^{DT}CCS_{He}$ of full-length UVR8 monomer.

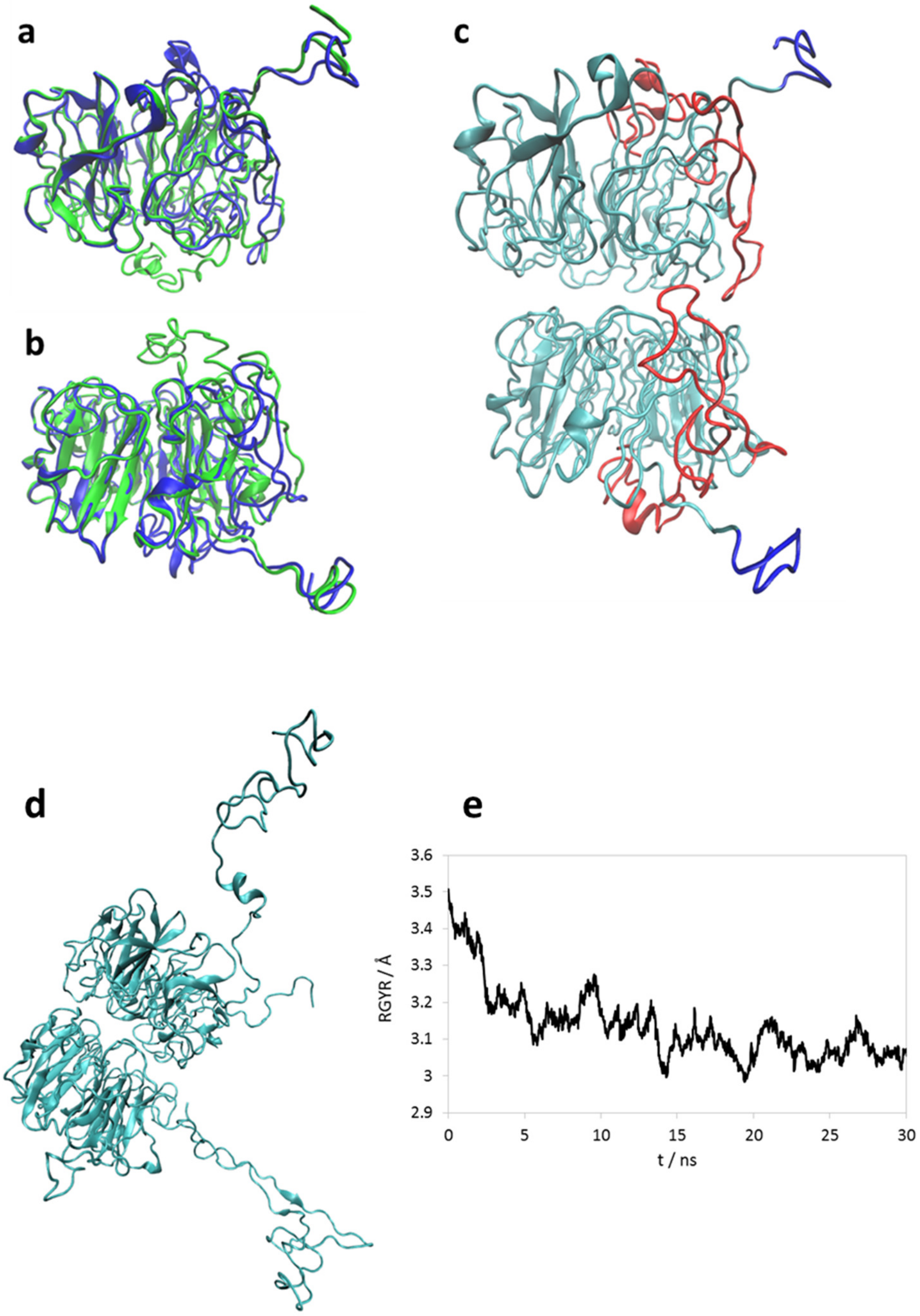

Figure S7. UVR8 model based on I-tasser models of the N- and C-terminus loops of the two chains from PDB 4dnw. The first model for each chain (**a**-**b**, green) placed the C-terminus loop such that they would clash in the dimer. The second model for each chain (**a**-**b**, blue) were therefore used to create the dimeric structure (**c**), with the N- and C- terminus loops shown in blue and red, respectively. Extended conformations were generated by pulling the centre of mass of the C-terminus from the core monomers (initial extended structure, panel **d**) and then allowing to relax (radius of gyration during relaxation, panel **e**).

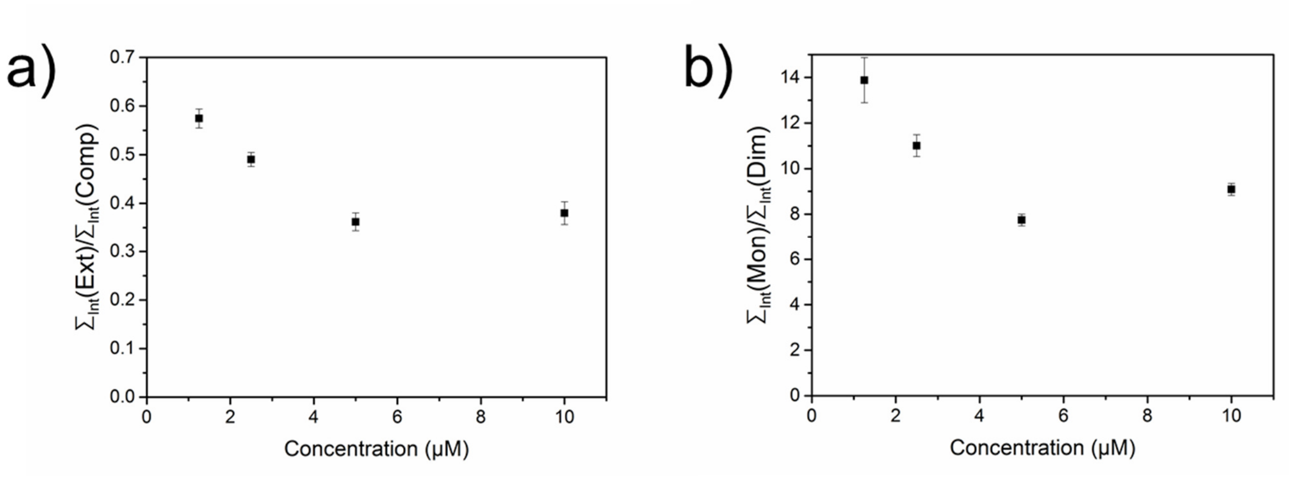

Figure S8. Concentration-dependence of full-length UVR8 conformations. **a)** Ratio of extended to compact dimers calculated from the combined signal intensities (Σ_{int}) as a function of protein concentration. At lower protein concentrations, the amount of extended dimer increases. **b)** Ratio of compact monomer to compact dimer after 10 s of exposure to the 280 nm LED as a function of protein concentration. At lower protein concentrations less dimer remains. This suggests a possible inner filter effect: the UVR8 monomer also absorbs 280 nm, thus 'shielding' a sub-population of dimers from UV-B exposure at higher concentrations.

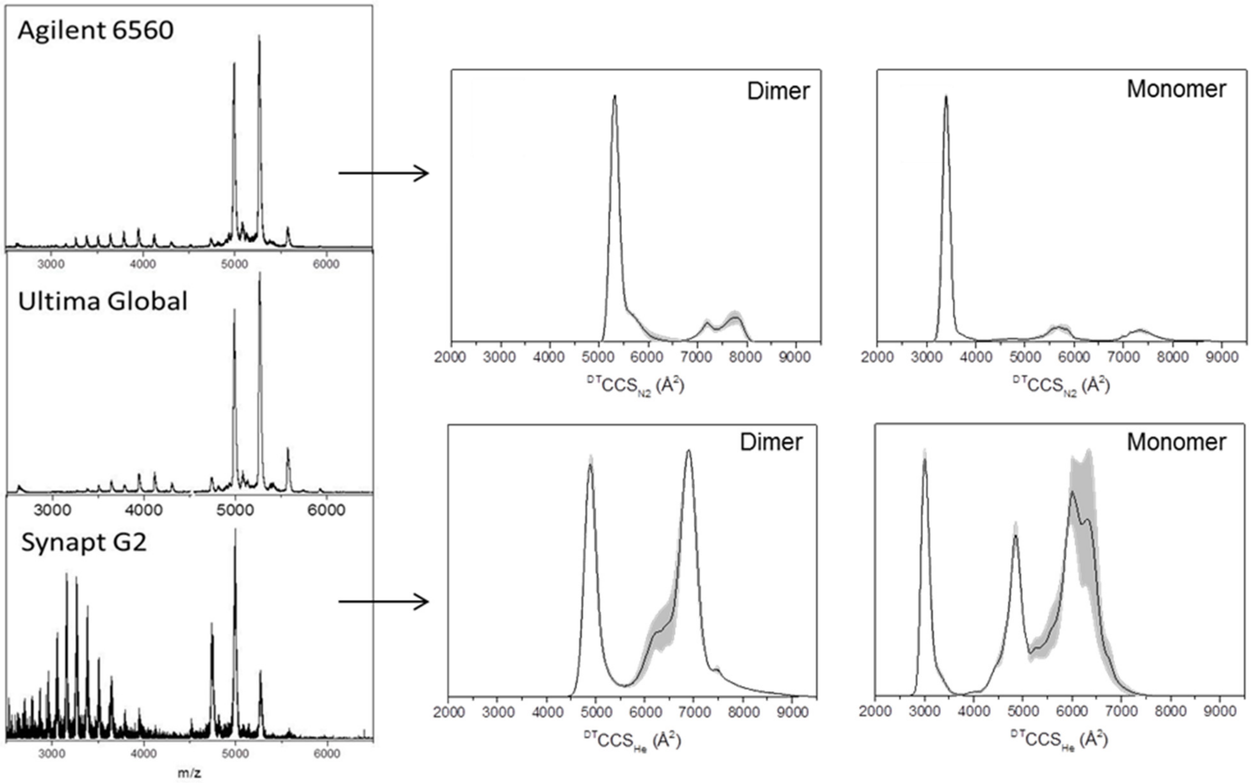

Figure S9. Native mass spectra of full-length UVR8 measured on three different instruments. All mass spectra (left hand panels) show a compact and a more extended species; however, the ratios between them vary. Ion mobility measurements (right hand panels) show that the conformational families remain the same across instruments and that it is only the intensities that vary.

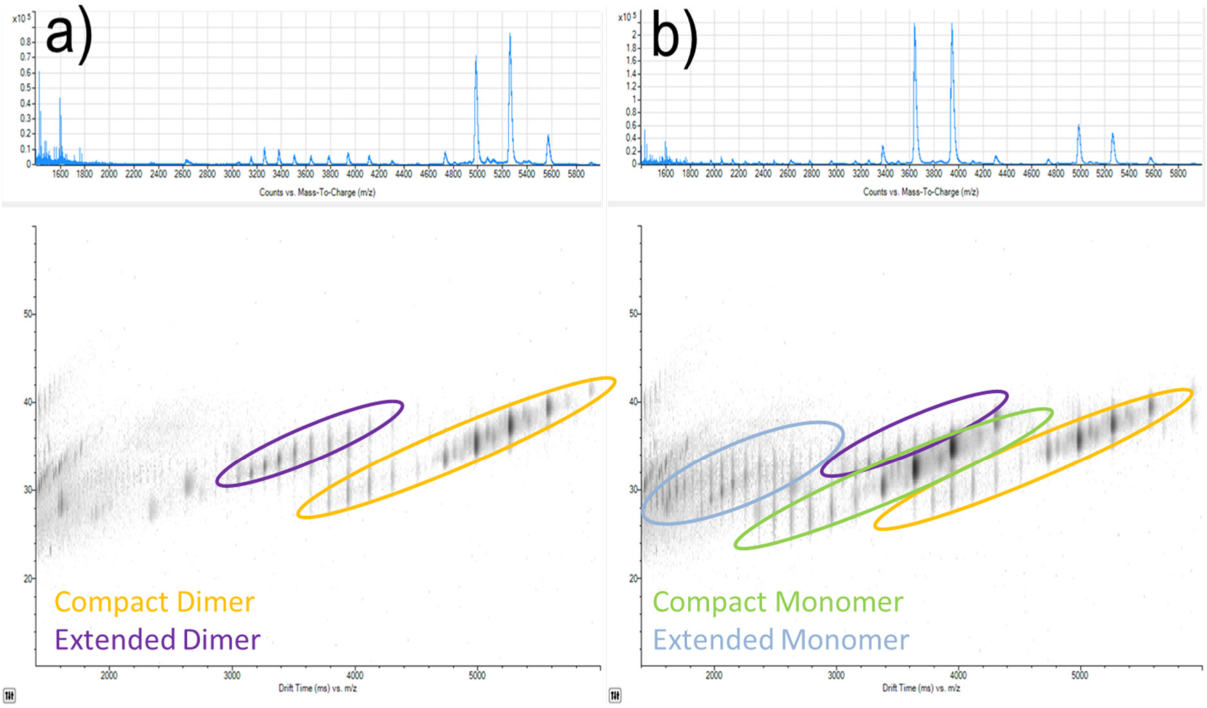

Figure S10. Ion mobility data of full-length UVR8 acquired on the Agilent 6560. **a)** The top panel shows the mass spectrum pre-irradiation, with the corresponding mobility heatmap underneath showing the drift times for each m/z. **b)** The top panel shows the mass spectrum after 1 s of 280 nm exposure, with the corresponding mobility heatmap underneath showing the drift times for each m/z. The ion mobility data allow discrimination between dimer and monomer where they coincide on the same m/z as is the case for the extended dimer and compact monomer. It is clear that the compact and extended dimers can still be seen post-irradiation, albeit at a much lower intensity than pre-irradiation. UV-B exposure has no impact on the measured $^{DT}CCS_{He}$ of the residual dimer conformations, and even extended periods of irradiation do not fully deplete their signals. Lowering the protein concentration does decrease the amount of dimer remaining (Figure S8b), however.

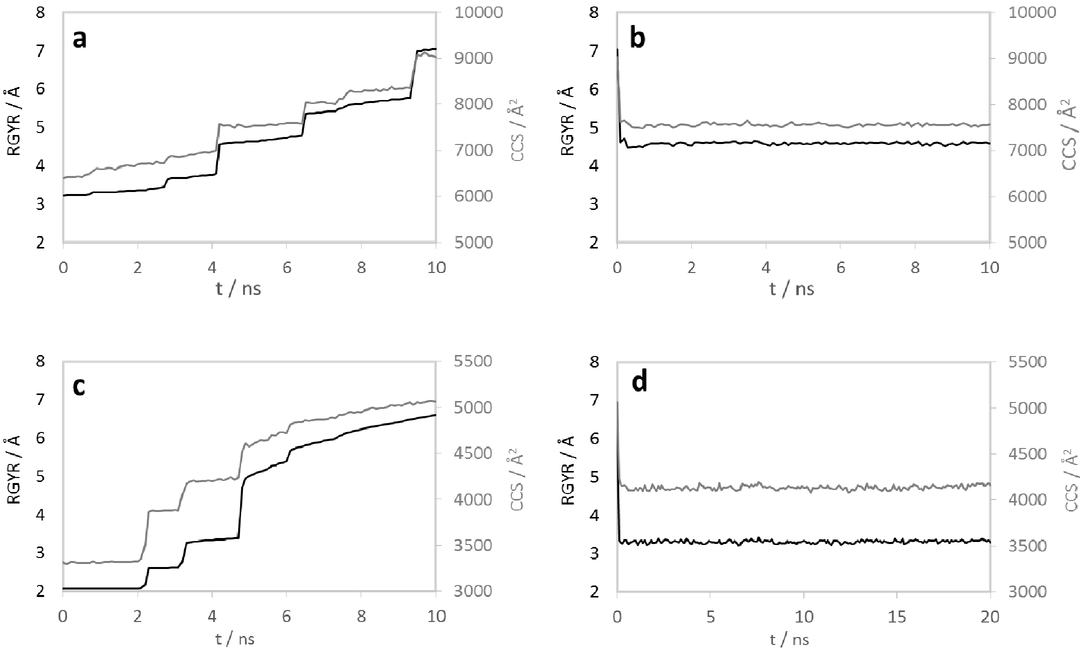

Figure S11. MD of full-length UVR8 following *in silico* extension of the C-terminal tails. Radius of gyration and computed CCS values for the dimer (**a**-**b**) and monomer (**c**-**d**) as the C-terminus is pulled into a fully-extended state (**a**,**c**) and subsequently relaxed (**c**,**d**).

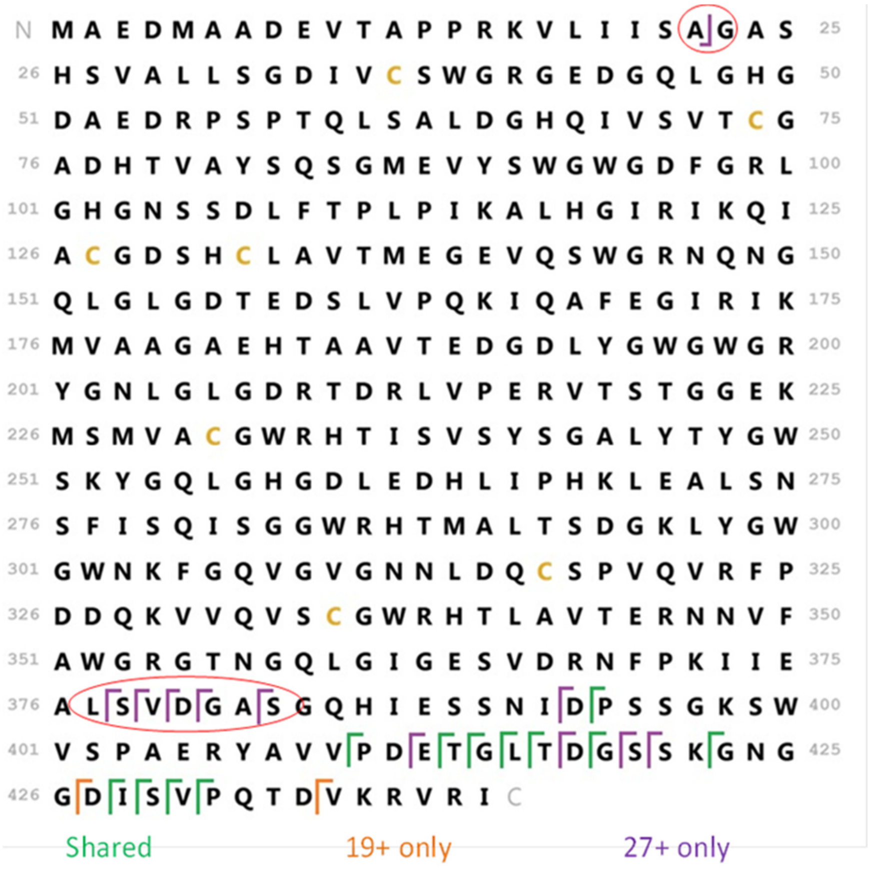

Figure S12. The UVR8 primary sequence illustrating the fragments observed upon collision-induced dissociation (CID). Fragments for the 19+ compact dimer are indicated in orange, the extended 27+ dimer in purple, and fragments found in the CID spectra of both in green. Fragmentation for both species occurs mainly in the C-terminal tail region; however, the extended dimer fragments more extensively. Loss of the N- and C-termini are only observed from the extended dimer (at the sites circled in red).

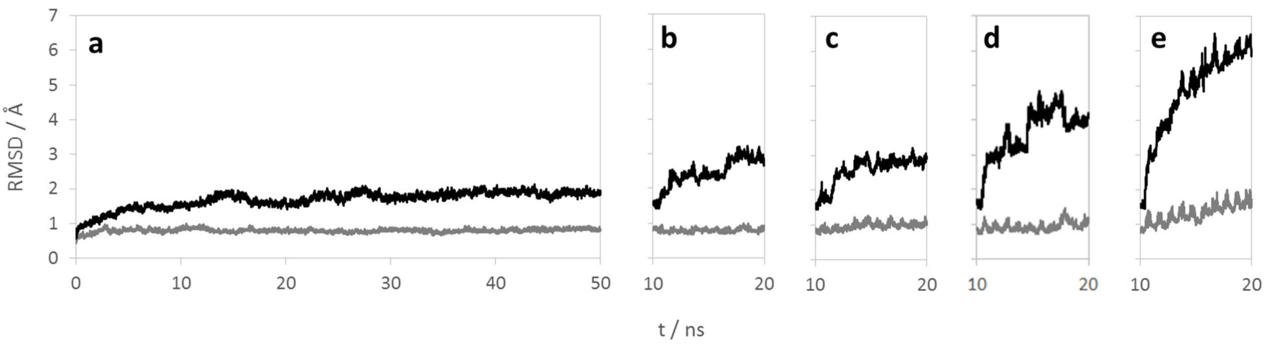

Figure S13. Root-mean-square deviation (RMSD) of backbone atoms for the core structure (residues 13 to 381) of the solvated full-length monomer (black lines) and core monomer grey lines during (**a**) 300 K MD simulations and (**b-e**) simulated annealing (*i.e.*, simulated unfolding) with 1 ns cycles between temperatures T_1 and T_2 as follows: (b) T_1 = 300 K, T_2 = 400 K; (c) T_1 = 350 K, T_2 = 425 K; (d) T_1 = 350 K, T_2 = 450 K; (e) T_1 = 350 K, T_2 = 500 K; see the Methods section for full details. Figure 5e shows each structure at the end of this annealing simulation. The structures shown in Figure 5e correspond to the end of the harshest simulation (**e**).

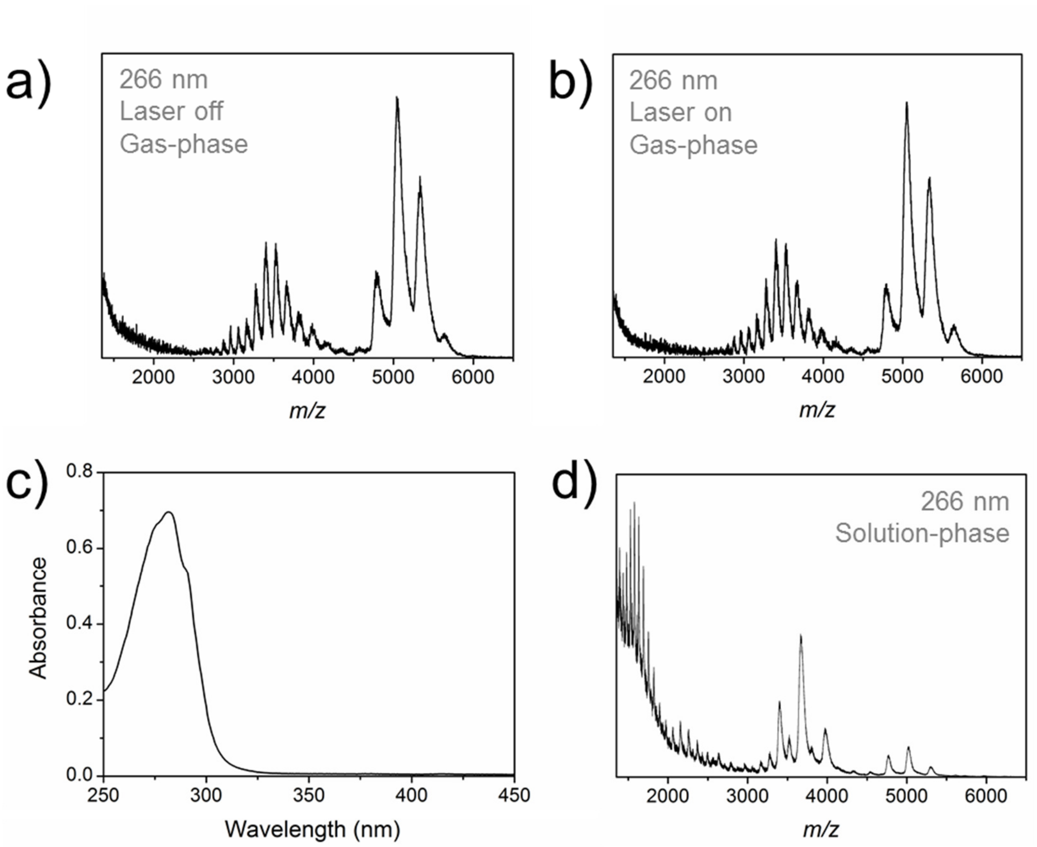

Figure S14. Native mass spectra of UVR8 before (**a**) and after (**b**) exposure to 266 nm laser pulses from the forth harmonic of an Nd:YAG laser in the gas phase. Data were collected using a modified Synapt G2-S (3). There is little or no discernible difference between the two spectra, suggesting that UVR8 does not appreciably convert to monomers in the gas phase even under illumination. **c)** UV-visible spectrum of UVR8 showing significant absorption at 266 nm. **d)** Native mass spectrum of UVR8 following exposure to 266 nm laser pulses in the solution phase, revealing the depletion of the dimer signal, accumulation of the monomer signal and therefore confirming that 266 nm photons can activate UVR8 in solution.

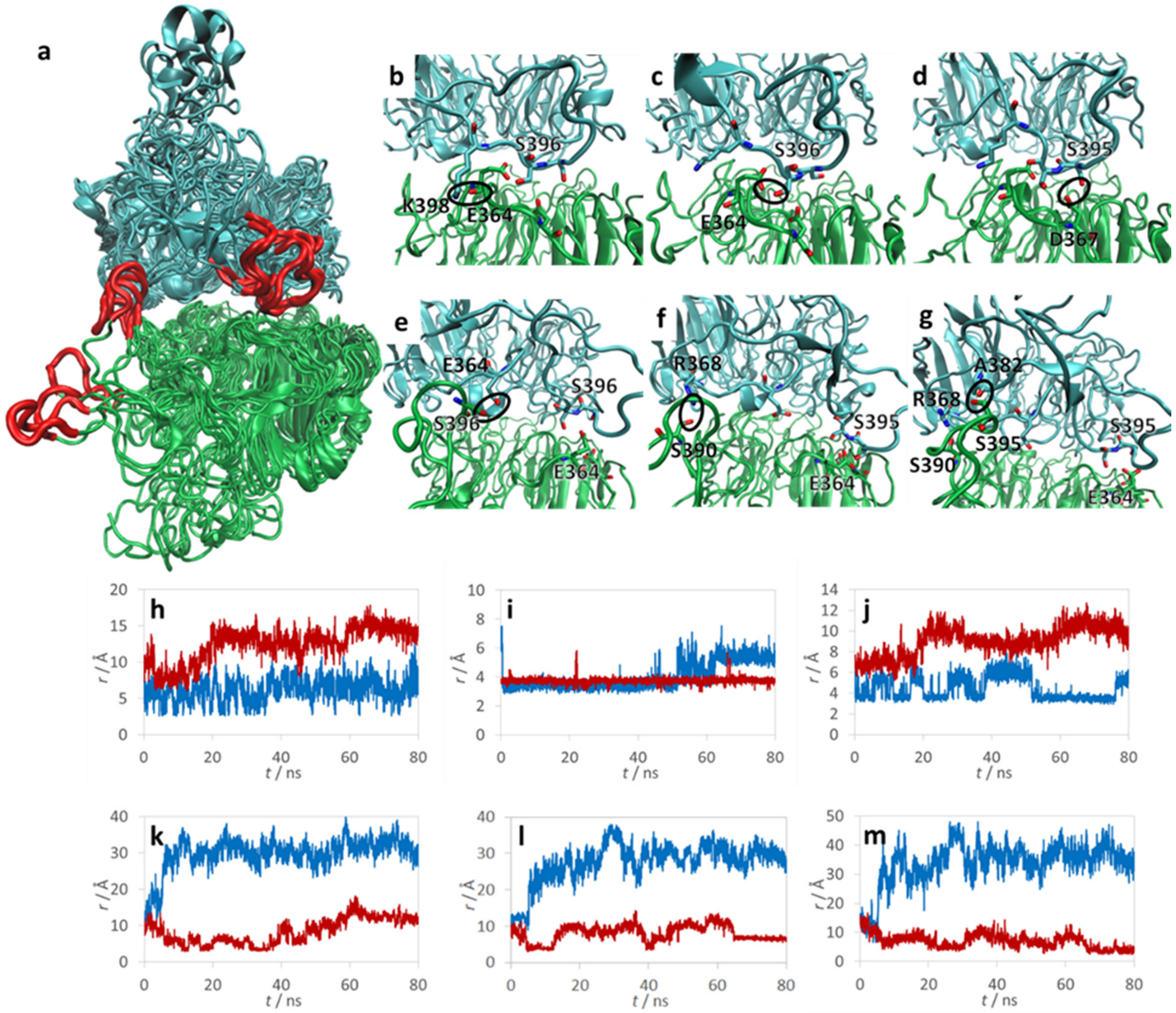

Figure S15. Example patterns of hydrogen bonding between the C-terminal loops of one monomer with the core domain of the opposite monomer from MD simulations. **a)** An overlay of structures with the six most populated H-bond patterns, which involve residues within the C-terminal region 390 to 398 (red). Three of the six patterns have both C-termini bound to the opposite monomer in a compact conformation, whereas the other three have one C-terminus bound and the other C-terminus unbound in an extended conformation. **b-g)** Example H-bond patterns between one C-terminal loop and the opposite monomer, where all residues involved in H-bonding are labelled. The specific H-bonds highlighted with black ovals in panels b-g correspond to a distance *vs* time plot for the heavy-atom distances (**h-m**, respectively). Two different MD simulations using different starting velocities were run in order to sample more of conformational space (see methods section for details), the results of which are plotted in blue and red, respectively, in panels h-m.

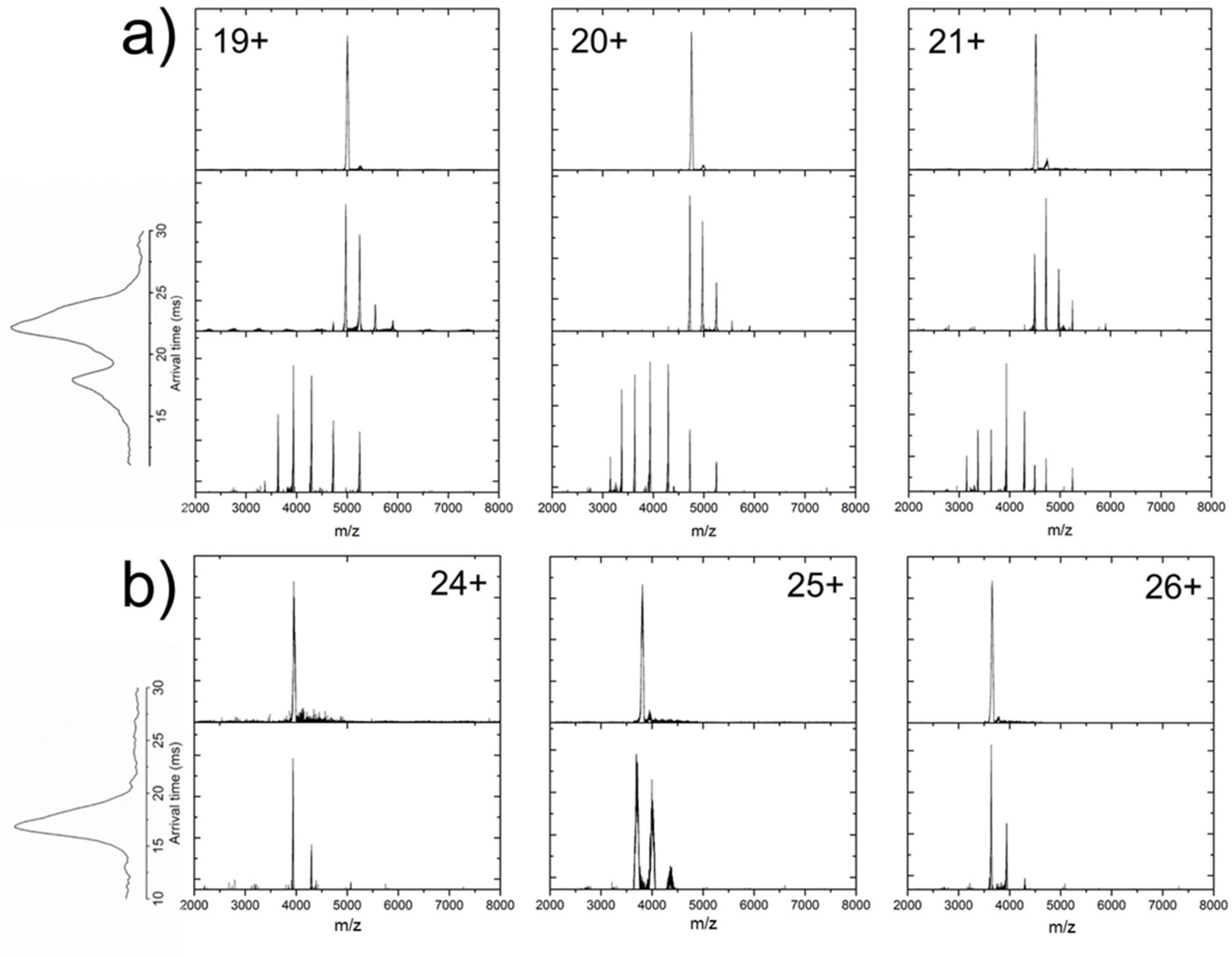

Figure S16. Surface-induced dissociation (SID) of the wild-type UVR8 dimer. **a)** SID of the compact charge states 19+, 20+ and 21+. The mass selected precursors are shown in the top panels. Following SID, two arrival time distributions are observed in the ion mobility data. The slower one corresponds to a charge stripped precursor shown in the middle panels, which is most likely due to the ion interacting with a layer of electrons on the gold surface target, and the faster one corresponds to monomeric units resulting from SID. Many charge states of the monomer are produced, which is termed an asymmetric dissociation. **b)** SID of the extended charge states 24+, 25+ and 26+. Mass selected precursors are shown in the top panels. Following SID, only a single arrival time distribution is observed that corresponds to a narrow charge state range of the monomeric unit, termed a symmetric distribution.

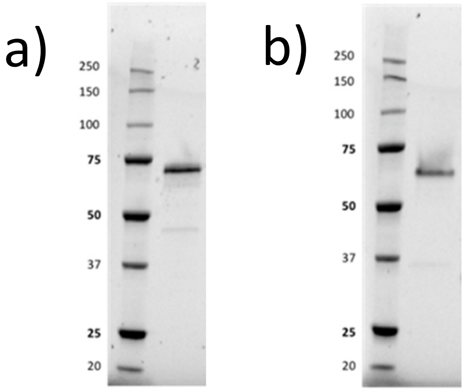

Figure S17. SDS-PAGE of purified full-length UVR8 **(a)** and UVR8$^{12\text{-}381}$ **(b)**. In both cases the molecular masss (kDa) marker is Precision Plus Protein™ Unstained Standards (Bio-Rad), the main, upper band is dimeric protein and the faint, lower band is a small amount of monomer.

References

1. Hanson J, Yang Y, Paliwal K, & Zhou Y (2017) Improving protein disorder prediction by deep bidirectional long short-term memory recurrent neural networks. *Bioinformatics* 33(5):685-692.
2. Christie JM*, et al.* (2012) Plant UVR8 Photoreceptor Senses UV-B by Tryptophan-Mediated Disruption of Cross-Dimer Salt Bridges. *Science* 335(6075):1492-1496.
3. Bellina B*, et al.* (2014) UV photodissociation of trapped ions following ion mobility separation in a Q-ToF mass spectrometer. *Analyst* 139(24):6348-6351.
